# The *yxiD* gene from *Bacillus subtilis* 6633 encodes a polymorphic toxin with tRNase activity and is neutralized by the cognate immunity protein YxxD

**DOI:** 10.1093/nar/gkaf1321

**Published:** 2025-12-12

**Authors:** Rishita Rohilla, Soni Kaundal, Krishan Gopal Thakur

**Affiliations:** Structural Biology Laboratory, CSIR-Institute of Microbial Technology, Chandigarh 160036, India; Academy of Scientific and Innovative Research (AcSIR), Ghaziabad 201002, India; Structural Biology Laboratory, CSIR-Institute of Microbial Technology, Chandigarh 160036, India; Structural Biology Laboratory, CSIR-Institute of Microbial Technology, Chandigarh 160036, India; Academy of Scientific and Innovative Research (AcSIR), Ghaziabad 201002, India

## Abstract

Polymorphic toxin systems (PTS) are widespread and play an important role in bacterial competition and shaping communities. However, these systems are less studied in Gram-positive bacteria. Here, we structurally and functionally characterized YxiD-YxxD, a predicted member of PTS from *Bacillus subtilis* 6633. Using growth curve assays, we established that YxiD-YxxD codes for a toxin-immunity protein pair. We determined a 1.7 Å resolution crystal structure of the YxiD^CTDH528A^ (*C*-terminal domain of YxiD) toxin bound to its cognate immunity protein YxxD. Structure revealed that the toxin adopts a Barnase/EndoU/colicin/RelE (BECR) fold, a characteristic of RNase toxins and the immunity protein sterically occludes binding of the molecular substrate to neutralize the toxin. Structural and other biophysical studies revealed that YxiD^CTD^-YxxD forms a stable 1:1 stoichiometric complex with K_D_ of ∼9.4 nM. RNA Sequencing experiments revealed that expression of toxin results in downregulation of several tRNAs and essential genes involved in cell wall biosynthesis, resulting in cellular toxicity. We further demonstrate that YxiD^CTD^ is a metal ion-dependent tRNase that cleaves several tRNAs. Taken together, our study provides the structural basis for YxiD neutralization by cognate immunity protein YxxD and establishes YxiD^CTD^ toxin as a metal-dependent tRNase.

## Graphical Abstract

**Graphical Abstract:**
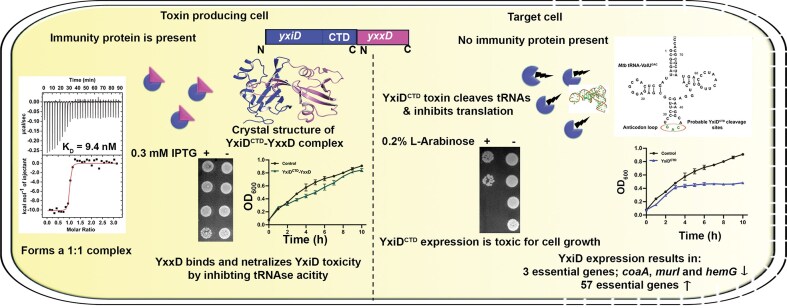

## Introduction

Microorganisms compete for limited resources and have developed defensive mechanisms to kill or cause growth arrest in their competitors. These systems help organisms gain an edge in resource utilization, niche colonization, and protection against threats, making them a fascinating area of research [1, 2]. One such biological defense mechanism is mediated by polymorphic toxin systems (PTS) that are widespread in bacteria [3–5] and were first comprehensively characterized in 2012 by Zhang *et al.*, who described their diverse architecture, secretion mechanisms, and ecological roles [5]. PTS play a crucial role in interbacterial competition and are often used as a defense against competitors.

While these systems have been extensively characterized in Gram-negative bacteria [1, 6, 7], recent studies have revealed that Gram-positive bacteria also employ polymorphic toxins to influence microbial communities [2, 8]. PTS encodes colicins [3, 9, 10], contact-dependent growth inhibition (CDI) systems [1, 11–16], RHS (Rearrangement hotspots toxins) [17–19], and some effectors of type VI [20–23] and VII secretion systems [24]. CDI systems are widespread in Gram-negative bacteria [25] and were first described in *Escherichia coli* EC93, where the *cdiBAI* operon mediates direct cell-cell growth inhibition through delivery of toxic CdiA *C*-terminal (CdiA-CT) domains into neighboring cells [26]. Known mechanisms of CDI-mediated growth inhibition include tRNase activity in CdiA-CT^E478^, cleaving specific tRNAs and arresting protein synthesis [27]; CdiA-CT^ECL^ cleaves 16S rRNA activity, cleaving ribosomal RNA to disrupt ribosome integrity [28]; CdiA-CT_II_^Dd3937^ from *D. dadantii* 3937 has Mg^2+^-dependent DNase activity, leading to DNA degradation and replication arrest [29]; and pore-forming toxins [30]. The toxin consists of two domains: The *N*-terminal domain that is involved in the export/trafficking of the protein and the *C*-terminal domain that harbors the toxic/effector module [3, 5]. The sequence of the *N*-terminal LXG (Leu-X-Gly) domain is generally conserved across bacterial species, but the smaller *C*-terminal domain exhibits substantial sequence and functional variability even between closely related strains [5, 31]. These *C*-terminal domains exhibit diverse toxic activities such as DNase, RNase, pore-forming activities, etc. [8, 30, 32]. These toxins are typically associated with an adjacent immunity-protein gene, which provides immunity to the host bacterium, preventing autoinhibition [1, 5]. The LXG domain is a conserved *N*-terminal domain found in polymorphic toxins, predominantly in Gram-positive bacteria such as *B. subtilis* [33]. The LXG domain plays a crucial role in the secretion of the toxic effectors across bacterial membranes by interacting with the Type VII secretion system (T7SS) [5, 31, 33–35].

A recent study has provided detailed structural insights into the mechanism of secretion of LXG domain toxin mediated by Type VII Secretion System (T7SS), which is predominantly found in Gram-positive bacteria [24]. The secreted toxin reaches a neighboring bacterial cell that expresses a receptor, often a bacterial surface protein like BamA [36] or OmpC in Gram-negative bacteria [37]. Although few structures of Gram-positive PTS have been reported [8, 38, 39], their structural and mechanistic understanding remains limited compared to Gram-negative counterparts, leaving a gap in our understanding of these modules. In *B. subtilis* 168, the existence of PTS was initially demonstrated through the identification of toxin domains such as YobL-CT, YxiD-CT, and YqcG-CT, which were shown to possess RNase activity [32]. In addition to these, YeeF/YezG constitutes a toxin-immunity protein pair, with YeeF functioning as a metal-dependent DNase [8, 38]. These systems highlight the diversity of antibacterial effectors in *B. subtilis*. The YxiD-YxxD toxin-immunity pair plays a significant role in interspecies competition among *B. subtilis* strains, particularly within biofilm communities [33]. Studies have demonstrated that strains lacking the *yxiD-yxxD* operon are outcompeted by wild-type strains, highlighting the importance of this system in competitive interactions [32]. In *B. subtilis* 168, the presence of PTS, including the LXG toxin YxiD and its immunity protein YxxD, has been well documented [32]. The study by Holberger *et al.* (2012) identified that the *C*-terminal domains (CT) of proteins from the PFam PF04740 family, including YxiD-CT from *B. subtilis* 168, exhibit cytotoxic ribonuclease (RNase) activity when expressed in *E. coli*, leading to growth inhibition. This cytotoxic effect is neutralized by cognate immunity proteins encoded downstream, such as YxxD, which specifically binds to YxiD-CT and inhibits its activity [32].

Considering the functional diversity in toxin modules [1, 8, 11, 32] it is crucial to characterize these diverse toxins to identify their molecular targets and the mechanism of bacterial growth inhibition.

In this study, we structurally and functionally characterized one of the members of the PTS from *B. subtilis* 6633 and confirmed that YxiD^CTD^-YxxD codes for a toxin-immunity protein pair. Interestingly, while the *N*-terminal domain is conserved, the *C*-terminal toxin domain shares only 23% sequence identity with *B. subtilis* 168 YxiD-CT. We report the crystal structure of the *C*-terminal domain of the toxin bound to its cognate immunity protein to reveal the mechanism of toxin neutralization. Additionally, detailed biophysical and biochemical studies were done to determine the binding stoichiometry and affinity of the toxin-immunity protein complex. RNA Sequencing (RNA-Seq) experiments were performed to understand the mechanism of growth inhibition and identify differentially expressed genes. We identified the molecular target of YxiD^CTD^ as tRNA, identified several substrate tRNAs and key residues required for activity/binding of the substrate. Taken together, these findings provide the structural basis for toxin neutralization by cognate immunity protein, establish the molecular target of the toxin, and provide the mechanism of growth inhibition.

## Materials and methods

### Construct design and engineering

The genes encoding *yxiD* (locus_tag: EO946_19 960) and *yxxD* (locus_tag: EO946_19 955) were PCR-amplified from *B. subtilis* 6633 (*Bacillus subtilis* subsp. *spizizenii* ATCC 6633) genomic DNA. The resulting PCR products were cloned into the pETDuet-1 vector (Novagen) between BamHI and EcoRI restriction sites. In addition, the *C*-terminal (Pro428-Pro534) domain of the *yxiD* gene encoding the toxin module, hereafter referred to as YxiD^CTD^, was cloned into an engineered version of pETDuet-1 (designated pETDuet-N, where the BamHI site was mutated to an in-frame NheI site) and into the pBAD vector using NheI and HindIII restriction sites with a *N*-terminal 6X His-tag to aid purification. Active site or substrate binding mutants of YxiD^CTD^ were generated through site-directed mutagenesis. It involved designing a single gene-specific primer that contained the desired nucleotide(s) substitution. YxiD-FL (full length) construct was cloned into the pET-22b vector between sites NcoI and XhoI with an *N*-terminal signal sequence for periplasmic expression. All the constructs were confirmed by DNA Sequencing, and no undesired mutations were observed. A comprehensive list of constructs generated and primers used in this study is provided in the Supplementary Table 1.

### Recombinant protein expression and purification

For protein purification, the pETDuet-1-YxiD^CTD^-YxxD construct was transformed into *Escherichia coli* Rosetta (DE3) cells (Novagen). A single colony was used to inoculate 10 mL of Luria–Bertani (LB) medium and cultured overnight at 37°C. Subsequently, 8 mL of the overnight culture was transferred into 800 mL of fresh LB medium and grown at 37°C until the optical density at 600 nm (OD_600_) reached approximately 0.6. Protein expression for pETDuet constructs was induced with 0.3 mM isopropyl β-D-1-thiogalactopyranoside (IPTG) (MP Biomedical), while for pBAD constructs, induction was performed using 0.2% L-arabinose (Sigma-Aldrich). Following induction, cultures were incubated for an additional 3 h at 37°C, harvested by centrifugation at 7000 g for 10 min at 4°C, and the resulting cell pellets were resuspended in lysis buffer containing 20 mM 4-(2-hydroxyethyl)-1-piperazineethanesulfonic acid (HEPES, pH 7.5) and 150 mM NaCl. Protease inhibitor cocktail tablets (Roche) were added to the lysis buffer prior to sonication to prevent proteolytic degradation. The lysate was clarified by centrifugation at 18 000 g for 40 min at 4°C. The supernatant was loaded onto a pre-equilibrated HIS-Select Ni-nitrilotriacetic acid (Ni-NTA) affinity resin (Sigma-Aldrich) for purification of the 6 × His-tagged protein. Bound proteins were eluted using a stepwise gradient of imidazole concentrations (20, 50, 100, 200, and 500 mM) prepared in lysis buffer. Eluted fractions containing the protein of interest were concentrated using centrifugal ultrafiltration devices with a 3 kDa molecular weight cut-off (Merck-Millipore). Further purification was performed by size-exclusion chromatography (SEC) using a Superdex 200 Increase 10/300 GL column (GE LifeScience) equilibrated in lysis buffer, at a flow rate of 0.5 mL/min. Protein elution profiles were monitored by absorbance at 280 nm, and fractions corresponding to the target protein were pooled and concentrated using 3 kDa ultrafiltration devices. The purity of the final protein preparation was assessed by SDS-PAGE, and the protein concentration was determined using the bicinchoninic acid (BCA) protein assay (ThermoFisher Scientific). All other variants (YxiD^CTD^, YxxD, YxiD^CTD^–YxxD complex, and active site mutants YxiD^CTDH528A^, YxiD^CTDH521A^, and YxiD^CTDH447A^, YxiD^CTDK446A^, and YxiD^CTDK524A^ were also purified using the above protocol. YxiD^CTD^ was expressed in *E. coli* Rosetta (DE3) cells using the pBAD expression system and subsequently purified. The pBAD system provides tight transcriptional control with minimum basal expression in the absence of arabinose, thereby preventing toxic protein accumulation during the preinduction cell growth stage. For large scale protein production, cultures were scaled up to 6-8 L, induced with 0.2% (w/v) L-arabinose, and incubated for 3 h at 37°C before harvesting for protein purification. The protein yield obtained was 6 mg/mL.

To perform RNase activity, proteins were purified to ensure RNase-free conditions during protein purification. All buffers were prepared using Diethyl Pyrocarbonate (DEPC)-treated water. DEPC was added to distilled water and was then autoclaved twice to hydrolyze residual DEPC. All protein samples were purified using size exclusion chromatography as mentioned above. However, the size exclusion column was equilibrated using DEPC-treated buffer to maintain RNase-free conditions throughout the purification process. All buffers (lysis buffer containing 20 mM HEPES (pH 7.5) and 150 mM NaCl, 10X TBE) were prepared similarly using DEPC-treated water to ensure that buffers remain free from RNase contamination, which was critical for the integrity of RNA for further cleavage studies.

### Growth curve, serial dilution spotting, and CFU count

The constructs encoding YxxD, YxiD^CTD^–YxxD complex, YxiD^CTD^, and active site mutants YxiD^CTDH528A^, YxiD^CTDH521A^, and YxiD^CTDH447A^ (including lysine mutants YxiD^CTDK446A^ and YxiD^CTDK524A^, identified as substrate binding mutants), along with the empty vector control, were transformed into *E. coli* Rosetta (DE3) cells (Novagen). Transformed cells were plated onto Nutrient Agar plates (HiMedia, Maharashtra, India) containing appropriate antibiotics and incubated overnight at 37°C. A single colony was used to inoculate 10 mL of Luria–Bertani (LB) medium and cultured overnight at 37°C with constant shaking at 200 rpm. Subsequently, 1% of the overnight culture was used to inoculate 10 mL of fresh LB medium, and was grown until an OD_600_ of approximately 0.5 was reached. This culture was further diluted to an initial OD_600_ of ∼0.05–0.1 in 10 mL LB medium. At an OD_600_ of ∼0.2, protein expression was induced by adding either 0.3 mM isopropyl β-D-1-thiogalactopyranoside (IPTG) to pETDuet-1 and pETDuet-N constructs, and 0.2% L-arabinose to pBAD constructs. OD_600_ measurements were recorded every 40 min for a total of 10 h using the Bioscreen C automated system (Oy Growth Curves Ab Ltd., Finland).

For dilution spotting assays, cultures were collected 3 h post-induction, 10-fold serially diluted in LB medium. 10 µL of these diluted samples were spotted onto LB agar plates supplemented with ampicillin (100 μg·mL^−1^) and chloramphenicol (35 μg·mL^−1^). For CFU count experiments, 25 µL of each sample was spread onto LB agar plates and incubated overnight at 37°C. Colonies were counted manually to check cell viability. Data are representative of three biological replicates.

### Transmission electron microscopy (TEM)

*E. coli*. strain Rosetta DE3 cells expressing YxiD^CTD^ (both induced and uninduced), YxiD^CTD^-YxxD, YxiD^CTDH528A^, and a vector control were analyzed using TEM to assess morphological changes associated with toxin activity. Bacterial cell pellets were washed twice with 0.85% saline and fixed using 2.5% glutaraldehyde at 4°C. 20 µL of the fixed sample was applied onto glow-discharged carbon-coated 300 mesh copper grids (Ted Pella, Inc.). Grids were stained with 2% uranyl acetate solution using standard negative staining procedures prior to imaging. The grids were subsequently air-dried, mounted onto the specimen holder, and imaged using a JEOL JEM-2100 transmission electron microscope operated at a voltage of 200 keV.

### Crystallization and structure determination

Crystallization trials were performed manually using 96-well sitting-drop vapor diffusion plates using 1 µL protein (15 mg/mL in buffer 20 mM HEPES, pH 7.5, and 50 mM NaCl) with 1 µL of reservoir solution. Crystallization trials were carried out for both the native YxiD^CTD^-YxxD complex and the catalytically inactive YxiD^CTDH528A^-YxxD complex. However, crystal hits were obtained only for the YxiD^CTDH528A^-YxxD complex in the ProPlex^TM^ screen (Molecular Dimensions, United Kingdom) in a condition containing 0.1 M HEPES pH 7.5 and 25%w/v PEG MME 2K after 3 days, which did not diffract. Further optimizations were done, and diffraction quality crystals were obtained in the condition 0.1 M Tris, pH 8.0, and 30% PEG MME 2K after 10 days. The crystals diffracted to a resolution of 1.7 Å at the ID30B beamline at ESRF, France. The data were processed using XDS [40] and scaled using AIMLESS [41] in the CCP4 software suite [42]. The structure was solved by the molecular replacement method using ColabFold [43] model as a template in PHASER [44]. Iterative steps of model building were performed in COOT [45] and refinement using Phenix.Refine [46] resulted in R_work_/R_free_ of 0.1875/0.2237. DALI server (47)was used to find close structural homologs. The oligomeric state and interface interactions between YxiD^CTDH528A^ and YxxD were analyzed using PDBePISA online server (https://www.ebi.ac.uk/pdbe/pisa/) [48].

The purified YxiD^CTDH528A^-YxxD protein complex was screened for crystallization using commercially available screens from Hampton Research (USA) and Molecular Dimensions (UK).

### Isothermal titration calorimetry (ITC)

The binding kinetics between YxiD^CTD^ and YxxD were obtained using Malvern AutoITC, measured at 25°C, 30 injections of 1.2 μl each, with a spacing of 180 s. Both proteins were purified using the same buffer, 20 mM HEPES (pH 7.5) and 150 mM NaCl. 15 μM of YxiD^CTD^ protein (in the cell) was titrated with 150 μM of YxxD protein (in the syringe) with a constant stirring speed of 600 rpm. The reference power was kept at 10 µCal/sec. Titration of the buffer alone was used as a control experiment using the same parameters as mentioned above. The data were analyzed and fitted using the OneSite binding model in Origin 2016 (Origin Lab Corporation) to determine the binding affinity (K_D_), stoichiometry of binding (N), enthalpy change (∆H), and entropy change (∆S) associated with the complex formation. The data presented here are representative of two independent experiments.

### *In-vitro* transcription and purification of tRNA

The ends of tRNA genes are highly conserved and pose a challenge in cloning. To overcome this challenge, tRNA genes, along with ∼200 bp flanking regions, were amplified from *E. coli* MG1655 and *Mycobacterium tuberculosis* H37Rv genomic DNA to avoid non-specific amplification. *E. coli* tRNA genes were cloned between NheI and HindIII restriction sites into the pBAD vector, and *Mtb* ValU (tRNA-ValU^GAC^) was cloned into the pUC vector using a single restriction site NheI via Seamless Ligation Cloning Extract (SLiCE) [49–51]. This strategy helped us in getting specific tRNAs cloned readily. The clones were confirmed by DNA sequencing.

To prepare a template for *in vitro* transcription, the tRNA gene-specific forward primer was designed with a 5′ overhanging T7 promoter sequence, while the reverse primer was specific to the 3′ tRNA region. For *in vitro* transcription, ∼1–2 µg of purified DNA was used as a template and 50 units of T7 RNA polymerase (ThermoFisher Scientific) in a 40 µL reaction as per the manufacturer’s protocol (ThermoFisher Scientific). The transcribed RNA was subsequently purified using the ZR Small RNA^TM^ PAGE Recovery Kit (Zymo Research, Irvine, CA). The purified tRNA was refolded as described in [52].

### RNA cleavage assay

The RNase-free protein samples were prepared as described in the section “Recombinant protein expression and purification.” For RNA cleavage activity, 500 ng–1 μg of tRNA was incubated with 10 µM of the desired protein sample in a cleavage buffer containing 10 mM HEPES, pH 7.5, 15 mM KCl, 1 mM DTT, 10 mM MgCl_2_ at 37°C for 15 min in a total reaction volume of 10 µL. The RNA samples were mixed with 2X RNA loading dye (containing formamide, bromophenol blue, and xylene cyanol), denatured at 70°C for 10 min, and immediately placed on ice. A 10% denaturing Urea PAGE gel was pre-run at a constant voltage of 200V for 30 min in 1X TBE buffer to ensure uniform gel conditions before sample loading. Samples were resolved by electrophoresis at a constant voltage of 100 V in the same buffer until the dye front migrated to the bottom. Following electrophoresis, the gel was stained with ethidium bromide (EtBr) solution for 15 min and then destained briefly in 1X TBE buffer to reduce background fluorescence. The gel was visualized using a gel documentation system.

### RNA-sequencing

The constructs encoding YxiD^CTD^ and YxiD^CTDH528A^ were transformed into *E. coli* strain Rosetta DE3 (Novagen). A single bacterial colony was selected and cultured in 10 mL of LB medium, incubated overnight at 37°C with constant agitation at 200 rpm. Subsequently, 1 mL of the overnight culture was used to inoculate 100 mL of fresh LB medium in a shake flask. The culture was grown until the OD_600_ reached ∼0.2–0.3. The bacterial culture was then induced by adding 0.2% L-arabinose, and the culture was allowed to incubate at 37°C for 2 h. Cells were harvested by centrifugation at 10 000 g for 10 min at 4°C. Bacterial cell pellets for both YxiD^CTD^ and YxiD^CTDH528A^ samples were sent to MedGenome Labs Ltd. India to perform RNA-Seq experiments. Total RNA was extracted using the RNeasy Mini Kit (Qiagen, Cat# 74 104) as per the manufacturer’s instructions, followed by rRNA depletion. The raw data was processed by MedGenome Labs Ltd., India. Contaminant removal was performed using Bowtie2 (version 2.2.4). The raw sequencing reads were processed using Trimmomatic to remove low-quality reads and adapter sequences, ensuring high-quality data. To quantify the uniquely mapped reads, FeatureCount (version 1.5.2) was utilized. These uniquely mapped reads were subsequently used for differential gene expression analysis using DESeq2. To calculate the fold change, the ratio of normalized read counts between treated and control samples was determined. Genes were filtered based on the adjusted *P*-value (≤0.05), ensuring statistical significance. The distribution of log_2-_fold change values was examined and found to follow a normal distribution. Genes exhibiting a log_2_-fold change between 0.1 and 1 were considered statistically significant. Differential expression of genes (both upregulated and downregulated) was identified based on a *P*-value threshold of ≤0.05. Gene Ontology (GO) annotation for these differentially expressed genes was performed using the PANTHER database [53], allowing functional categorization and insights into the biological processes associated with the expression changes. The analysis of essential genes was performed manually using the BioCyc database [54].

### Gene expression validation using RT-qPCR

Reverse transcription quantitative real-time PCR (RT-qPCR) was performed to validate differentially expressed genes (DEGs) identified through RNA-Seq analysis, using gene-specific primers designed based on the RNA-Seq data for selected upregulated and downregulated transcripts. Total RNA was extracted from bacterial cultures expressing YxiD^CTD^ and YxiD^CTDH528A^ (technical replicates of RNA-Seq samples) using the GeneJET™ RNA Purification Kit (ThermoFisher Scientific), as per the manufacturer’s protocol. RNA integrity was assessed by electrophoresis on a 6% Urea–PAGE gel. RT-qPCR reactions were performed using the iTaq™ Universal SYBR Green One-Step Kit (Bio-Rad, Cat# 172–5151) in a final reaction volume of 10 µL according to the manufacturer’s protocol. Amplifications were carried out on a Bio-Rad CFX96 Real-Time PCR Detection System, and melt curve analysis was conducted to verify amplification specificity. The housekeeping gene *rpoD* was used as an internal reference for normalization. Relative gene expression levels were calculated using the 2^–ΔΔCt^ method, where ΔCt represents the difference between the cycle threshold (Ct) values of the target gene and the internal control and ΔΔCt indicates the fold change between treated and control samples.

### Computational modeling and molecular graphics visualization

Structural homologs were identified using the DALI server [47] (http://ekhidna2.biocenter.helsinki.fi/dali/), while sequence alignment was performed using MultAlin [55] to generate multiple sequence alignments (MSA). The alignment files were further processed using ESPript 3.0 [56] (https://espript.ibcp.fr/ESPript/ESPript/) and Jalview [57] (version 2.11.4.1) to create input files for the WebLogo server [58] (https://weblogo.berkeley.edu/logo.cgi), enabling visualization of conserved motifs. Conservation mapping on protein structures was carried out using the ConSurf web server [59]. PyMOL (Schrodinger, LLC. version 2.5.5) was utilized to generate structural figures. Graph generation and statistical analysis were carried out using GraphPad Prism (version 10.4.2). The tRNA schematic was generated using the R2DT tool available at RNA central [60] and subsequently edited using RNAcanvas [61].

## Results

### YxiD-YxxD is a toxin-immunity protein pair

**Figure 1.**
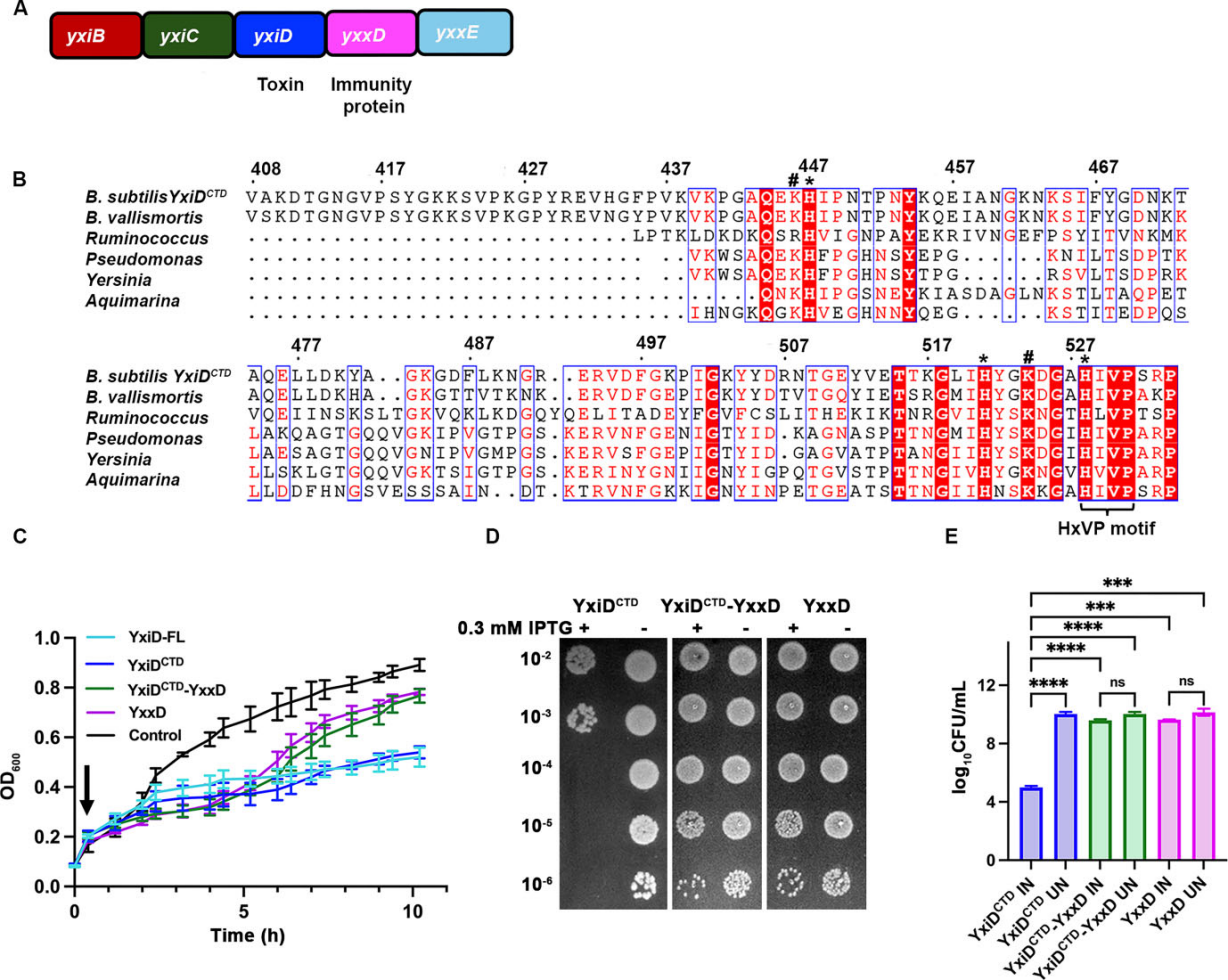
YxiD-YxxD codes for a toxin-immunity protein pair. **(A)** Schematic representation (not to scale) of the predicted operon consisting of the yxiD, yxxD, and other neighboring genes in *B. subtilis* 6633. yxiB, yxiC, and yxxE genes are part of the same operon, which are predicted to play a role in the secretion of YxiD. **(B)** Multiple Sequence Alignment (MSA) analysis of YxiD^CTD^ from different bacterial species showing the conservation of active site residues marked with an asterisk (*), hash (#) represents highly conserved positively charged residues K/R, and a common HxVP motif, consisting of His528, Ile529, Val530, and Pro531 of YxiD^CTD^. **(C)** Growth curve analysis showing the effect of toxin overexpression on bacterial growth. The black arrow indicates the induction point. Data represent mean ± s.d. from three independent experiments performed in technical triplicates (*n* = 3). **(D)** Dilution spotting analysis showing no colony formation after 3-fold dilution in toxin overexpressing cells. Serial dilutions (10^−2^ to 10^−6^) are indicated on the *y*-axis. Data are representative of three independent replicates (*n* = 3). **(E)** Colony Forming Unit (CFU) Count analysis representing the effect of YxiD^CTD^ on bacterial viability in treated samples compared to the control. (IN - induced samples, UN - uninduced samples). Data are representative of three independent experiments (*n* = 3). Expression of YxiD resulted in a 4.6 ± 0.6 log₁₀ reduction in CFU (mean ± SD, *n* = 3; *P* < 0.0001, unpaired t-test on log₁₀ CFU). Significance is indicated as ^****^ (*P* < 0.0001), *** (*P* < 0.001), and ns = not significant (*P* > 0.05).

We selected an annotated PTS system from *B. subtilis* 6633, consisting of a polymorphic toxin yxiD (Protein ID: QCY19253.1) and yxxD (Protein ID: QCY19252.1) immunity protein. The genes *yxiB, yxiC*, and *yxxE* are present adjacent to *yxiD* within the genome and are predicted to be involved in the secretion of YxiD^CTD^ via Type VII Secretion System (T7SS). This suggests an operon-like organization that includes proteins primarily involved in efficient expression and secretion of the toxin [34] (Fig. 1A). Multiple sequence alignment (MSA) analysis of YxiD^CTD^ revealed the presence of highly conserved histidine and lysine residues, along with a distinctive HxVP motif, indicating its potential catalytic role in the toxic activity (Fig. 1B). To assess the effect of YxiD^CTD^ expression on bacterial growth, we monitored the growth of *E. coli* expressing YxiD-FL (full-length toxin, excluding signal sequence), YxiD^CTD^, YxxD, and YxiD^CTD^-YxxD complex. After 10 h of incubation at 37°C, it was observed that cells expressing YxiD-FL and YxiD^CTD^ alone exhibited growth inhibition, confirming that YxiD^CTD^ functions as a toxin. As the *C*-terminal domain represents the toxic domain, all further experiments were performed using this construct. Interestingly, expression of YxxD alone resulted in a prolonged lag phase during the initial hours of growth. However, the cells eventually resumed exponential growth, indicating that YxxD itself is not toxic to the host. Co-expression of YxiD^CTD^ with YxxD restored growth, supporting the role of YxxD as a functional immunity protein that can neutralize the toxic effects of YxiD^CTD^ (Fig. 1C, Supplementary Table 2). Serial dilution spotting assay demonstrated that induction of YxiD^CTD^ led to a marked reduction in cell viability after 3-fold dilution, consistent with its toxic activity. In contrast, co-expression of YxxD with YxiD^CTD^ completely restored growth (Fig. 1D). So, these data establish that YxiD-YxxD forms a toxin-immunity protein pair.

To further evaluate YxiD^CTD^ toxin activity, CFU count experiments were performed to assess the effect of YxiD^CTD^ expression on cell viability. Samples were collected 3 h post-induction, and 10-fold serial dilutions of bacterial cultures were prepared in LB medium. Aliquots of 25 µL from each dilution were spread onto LB agar plates. Following incubation at 37°C overnight, colonies were counted to calculate CFU/mL. The expression of YxiD^CTD^ alone led to a significant reduction in viable cell count, with more than four log_10_ reduction confirming high toxicity. In contrast, co-expression of YxxD with YxiD^CTD^ restored CFU levels, confirming the role of YxxD in neutralizing toxin-induced cell death (Fig. 1E). These findings provide strong evidence that expression of YxiD^CTD^ is toxic for cells and co-expression of YxxD effectively neutralizes the deleterious effects of the toxin. This data further confirms that YxiD-YxxD encodes for a toxin-immunity protein module.

### Crystal structure of YxiD^CTDH528A^-YxxD complex

To gain further insights into the function of toxin and the mechanism of toxin neutralization by cognate immunity protein, we determined the crystal structure of the YxiD^CTDH528A^-YxxD complex at 1.7 Å resolution. The purified complex used for crystallization is shown in Supplementary Fig. S1. We have solved the structure of the active site mutant YxiD^CTDH528A^ in complex with the immunity protein, where highly conserved His528 has been substituted with alanine. The H528A substitution was introduced because His528 is highly conserved across multiple sequence alignments and is probably involved in catalytic activity. The structure was determined using a ColabFold [43] predicted model of the toxin-immunity protein complex. The protein-protein complex crystallized in the space group C222_1_ (a = 63.38 Å, b = 70.89 Å, c = 91.79 Å, α = 90°, β = 90°, γ = 90°). There was one copy each of toxin and immunity protein in the asymmetric unit. The structure consists of Pro428-Pro534 (107 aa residues) in YxiD^CTDH528A^, and the initial 20 residues, including His-tag, could not be resolved in the electron density, probably due to disorder. All the residues in YxxD from Gly2-Asp110 (109 aa residues) were resolved well in the electron density map. The detailed data collection and refinement statistics are provided in the Table 1.

**Table 1.** Data collection and refinement statistics

	YxiD^CTDH528A^-YxxD
**Data Collection**	ID23-2, ESRF, France
Resolution range (Å)^a^	43.11–1.7 (1.73–1.7)
Space Group	C222_1_
Unit cell parameters	
a, b, c (Å)	67.38, 70.89, 91.79
α, β, γ (°)	90°, 90°, 90°
No. of unique reflections^a^	23 814 (1235)
Average mosaicity (°)	0.05
Redundancy^a^	6.7 (6.8)
Average *I*/σ*I* ^a^	11.2 (2.8)
Completeness (%)^a^	97.6 (97.0)
R_merge_ (%)^a^	13.9 (58.2)
CC1/2^a^	0.98 (0.84)
**Refinement statistics**	
Resolution range (Å)	33.44–1.7
No. of reflections used in the refinement^a^	23 810 (2916)
R_work_ (%)	0.1875
R_free_ (%)	0.2237
r.m.s.d.^a#^	
Bond lengths (Å)	0.007
Bond angles (°)	0.99
Ramachandran plot statistics	
Most favoured (%)	98.10
Allowed regions (%)	1.90
No. of protein residues	215
No. of solvent atoms	403
Wilson B-factor (Å)	13.46
Average B-factor (Å^2^)	19.57
Protein atoms	17.39
Solvent atoms	28.97

a Values for the last shell are in parentheses.

a# r.m.s.d. root mean square deviation

**Figure 2.**
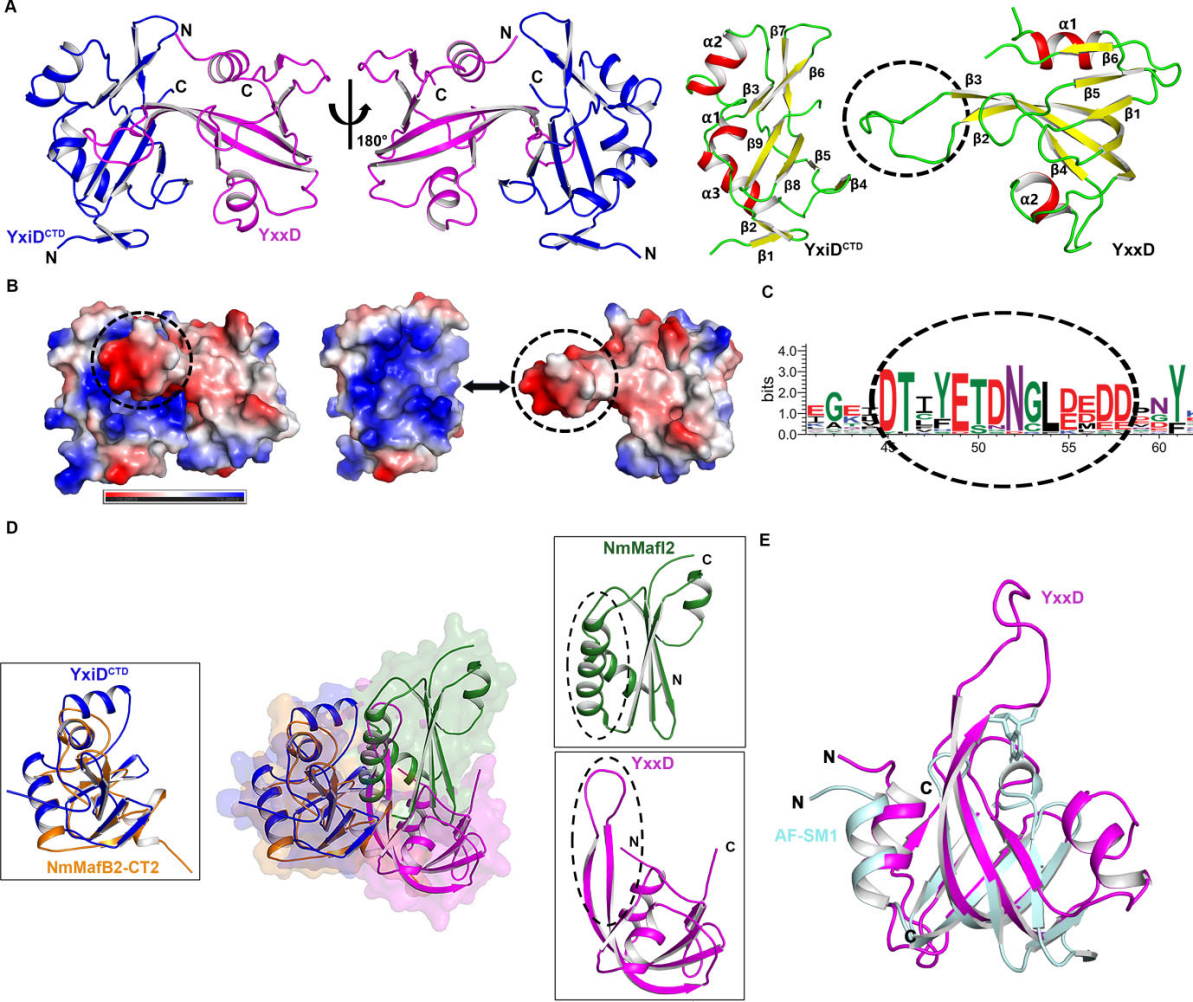
Crystal structure of YxiD^CTDH528A^-YxxD complex. **(A)** Cartoon representation of the YxiD^CTDH528A^-YxxD complex in two different orientations, shown in blue and magenta colors, respectively (left panel). Cartoon representation with labeled secondary structural elements α-helices (red), β-sheets (yellow), and loops (green) (right panel). **(B)** Electrostatic potential distribution of the complex. The dotted circle represents the negatively charged region in the immunity protein which binds the positively charged active site involved in binding RNA substrate and sterically occludes binding substrate, YxxD sterically occludes binding of substrate to neutralize the toxin. **(C)** WebLogo representation of the conserved negatively charged loop in YxxD involved in binding the positively charged active site of YxiD. **(D)** Structural superposition of YxiD^CTD^ and ribonuclease toxin NmMafB2-CT2 toxins alone (left panel) and YxiD^CTD^-YxxD complex with a ribonuclease toxin NmMafB2-CT2/NmMafI2 complex from *N. meningitidis* B16B6 (PDB: 8HHJ) [65] (middle panel). YxxD and NmMafI2 immunity proteins adopt different conformations and have different mechanisms of molecular recognition (right panel). (YxiD^CTDH528A^ - blue, YxxD - magenta, NmMafB2-CT2 - orange, and NmMafI2 - green color). **(E)** Superposition of YxxD with Sm-like protein (AF-SM1) present in *A. fulgidus* (PDB: 1I5l) [66].

YxiD^CTDH528A^ structure consists of three α-helices (α1–α3) and three antiparallel β-sheets, consisting of nine β-strands (β1-β2, β3-β6-β7, β4-β5-β8-β9) (Fig. 2A, right panel). The topology diagram is shown in the Supplementary Fig. S2. The α-helices are positioned adjacent to the beta sheet core. The structure is further stabilized by four β-hairpins connecting β-strands. The topology also highlights the presence of a conserved fold typical of RNase toxins, where the β-sheet core is crucial for forming the catalytic pocket. Crystal structure revealed that the YxiD^CTD^ adopts Barnase/EndoU/colicin/RelE (BECR) fold [5, 62], a characteristic of RNase toxins. The structural analysis using ProFunc [63] further suggested that YxiD^CTD^ belongs to PFam PF15542 (Ntox50) and contains three conserved histidines, likely critical for its catalytic function. We performed a homology search using the DALI server [47] and found that YxiD^CTD^ shares structural similarity with ribonucleases. Crystal structure revealed that the YxiD^CTD^ adopts Barnase/EndoU/colicin/RelE (BECR) fold [5], [62], a characteristic of RNase toxins. YxiD^CTD^ showed high structural similarity with a known RNase, NmMafB2-CT2 (PDB: 8HHJ) from *Neisseria meningitidis* B16B6 (r.m.s.d of 1.42 Å over 101 aligned Cɑ atoms and 32.67% sequence identity), indicating a strong structural conservation. Structural superposition of YxiD^CTD^-YxxD complex with NmMafB2-CT2-NmMafI2 complex from *N. meningitidis* B16B6 is shown in Fig. 2D. In contrast, other homologs such as RNaseI (PDB: 2VQ9: A), pancreatic ribonuclease from *Mus musculus* (PDB: 3TSR: D), and Ribonuclease A (PDB: 1RHA: A) displayed lower structural similarity, Z-scores around 4.4-4.5, r.m.s.d values of 2.9-3.0 Å, and sequence identities from 4% to 6%. These findings indicate that YxiD^CTD^ adopts a ribonuclease-like BECR fold, indicating a conserved structural framework potentially associated with its functional role.

The crystal structure revealed that YxxD consists of two α-helices (α1 and α2) and one β-sheet, comprising six antiparallel β-strands (β1–β6) (Fig. 2A). PDBePISA [48] analysis suggests that YxiD^CTD^-YxxD complex buries an area of 2100 sq. Å, suggesting a tight association. To investigate the evolutionary conservation of YxiD^CTD^ toxin, we performed ConSurf analysis [59]. The analysis revealed that the active site pocket of the toxin, including conserved Histidine residues that bind immunity and potential substrate binding residues, is evolutionarily conserved. (Supplementary Fig. S3).

Structural homology search for YxxD performed using DALI server [47] revealed structural similarity to Sm-like protein from *Archaeoglobus fulgidus* (PDB: 1I5L: G, r.m.s.d. 2.14 Å over 72 Cα atoms) with a low sequence identity of 14.08% only (Fig. 2E). Other structural homologs included YaeO from *Vibrio cholerae* (PDB: 6JIE: A, r.m.s.d. 2.6 Å over 77 Cα atoms), RNA chaperone or RNA-binding proteins such as Hfq protein from *B. subtilis* 168 (PDB: 3HSB: E, r.m.s.d. 2.7 Å over 68 Cα atoms) involved in post-transcriptional regulation, and the U6 snRNA-associated Sm-like protein Lsm8 from *Saccharomyces cerevisiae* (PDB: 4M77: B, r.m.s.d. 2.7 Å over 91 Cα atoms) [64] with sequence identities from 11% to 15%. These findings suggest that although YxxD adopts an Sm-like fold of RNA-binding proteins, it has diverged significantly in sequence and functional adaptations.

### YxxD sterically occludes substrate binding

The electrostatic potential distribution of the YxiD^CTDH528A^-YxxD complex structure reveals the presence of both positively and negatively charged regions on its surface. The structure revealed that the YxxD binds to the active site of the toxin, consisting of highly positively charged residues and sterically occludes binding of the substrate to neutralize the toxin. The electrostatic surface charge potential for both the toxin and the immunity protein shows a highly negatively charged region present on YxxD, which binds the positively charged active site of the toxin (Fig. 2B). Structure reveals that the loop connecting from (Asp33-Asp46) β2 and β3 of YxxD plays a key role in binding to the active site of the toxin (Fig. 2A, Right panel). This loop is highly negatively charged, composed predominantly of acidic residues (D/E) (Fig. 2B and C). This region serves as a crucial structural feature, facilitating the interaction by positioning key residues within the binding interface. The spatial arrangement of this region and its molecular interactions play an important role in molecular recognition, a functional determinant in toxin neutralization. Interestingly, while YxiD^CTD^ shares reasonable structural homology with NmMafB2-CT2 toxin (Fig. 2D, left panel), YxxD does not align with the NmMafI2 immunity protein, suggesting differences in the mechanism of toxin neutralization. While in YxxD, the blocking of the toxin’s active site is mediated by the β2–β3 loop, in NmMafI2, two alpha-helices are involved in the toxin neutralization, highlighting different modes of inhibition in these toxins (Fig. 2D, right panel). Fig. 2E shows the superposed structure of YxxD with the AF-Sm1 protein from *A. fulgidus* and reveals that the β2 and β3 extended loop region present in YxxD is absent in AF-Sm1. The presence of this loop may represent an evolutionary adaptation in YxxD to improve target recognition and binding specificity.

### YxiD^CTD^-YxxD form a 1:1 complex with a nanomolar range dissociation constant

**Figure 3.**
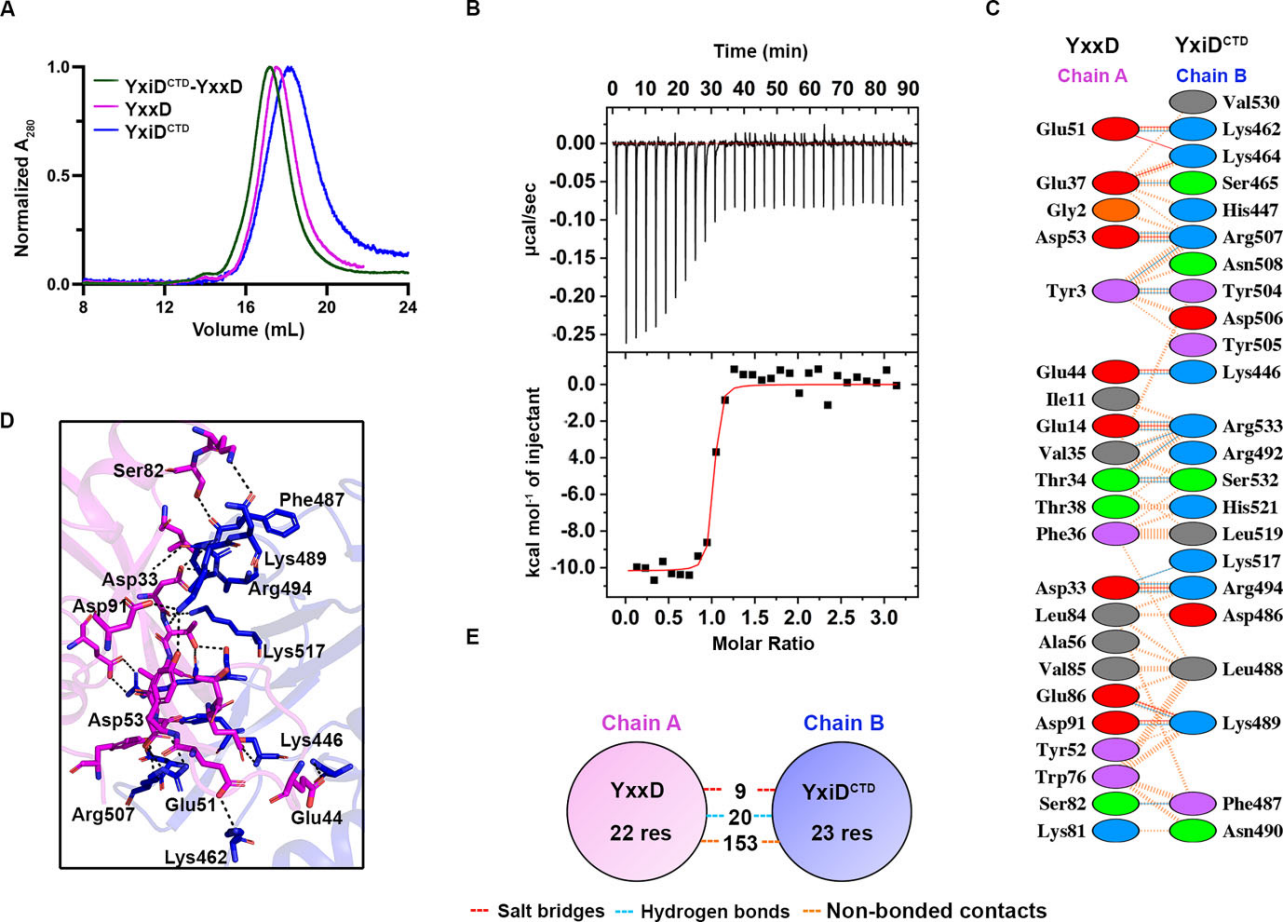
Binding studies and interaction network of YxiD^CTD^-YxxD complex **(A)** SEC analysis showing the overlay of chromatograms representing the elution profile of YxiD^CTD^ (blue), YxxD (shown in magenta), and YxiD^CTD^-YxxD (green) complex. **(B)** Isothermal titration calorimetry represents the thermodynamic analysis of the interaction between YxiD^CTD^ and YxxD. **(C)** Schematic representation of the key interacting residues, obtained by ProFunc analysis [63], involved in the protein-protein interaction interface. **(D)** Cartoon representation of the binding interface with polar residues shown in stick representation, visualized using PyMOL. Key interactions are shown in the dotted lines. **(E)** Schematic representation of the key interactions contributing to the stability of the complex.

Toxin needs to be neutralized effectively to minimize adverse growth effects to the host or producer cells. So, to determine the binding affinity and stoichiometry of YxiD^CTD^-YxxD complex, we performed size exclusion chromatography (SEC) and ITC experiments. When we mixed both the proteins YxiD^CTD^ and YxxD in equimolar ratios, we obtained a peak shift at 17.2 mL, suggesting that YxiD^CTD^-YxxD forms a complex (Fig. 3A). ITC experiments revealed that YxiD^CTD^-YxxD forms a tight complex with a K_D_ of ∼9.4 ± 5.8 nM, indicating a high-affinity complex. The data were fitted using the OneSite binding model, showing that the binding was associated with a favorable enthalpy change (∆H) -10170 ± 209.7 cal/mol, suggesting that the reaction is exothermic and is primarily driven by enthalpic contributions due to hydrogen bonding, salt bridges, and van der Waals interactions. The binding stoichiometry (N) of 0.975 ± 0.00978 sites suggests that YxiD^CTD^ forms a 1:1 stoichiometric complex with YxxD, as supported by the crystal structure (Fig. 3B).

Structural analysis using ProFunc [63] identified the key interacting amino acid residues involved in the complex formation. Specifically, residues such as Lys462, Ser465, Arg507, Lys446, Arg533, Arg494, and Lys489 (positively charged residues) present on the toxin interact with Glu51, Glu37, Asp53, Glu44, Glu14, Asp33, Glu86, and Asp91 (negatively charged residues) on the immunity protein (Fig. 3C, 3D). These residues are located primarily within the binding interface of both the toxin and immunity protein, engaging in nine salt bridges, 20 hydrogen bonds, and 153 non-bonded contacts (Fig. 3E). Taken together, the structural data and binding kinetics suggest that YxiD^CTD^-YxxD forms a high-affinity complex with a low nanomolar range dissociation constant.

### YxiD^CTD^ is a metal ion-dependent toxin with tRNase activity

**Figure 4.**
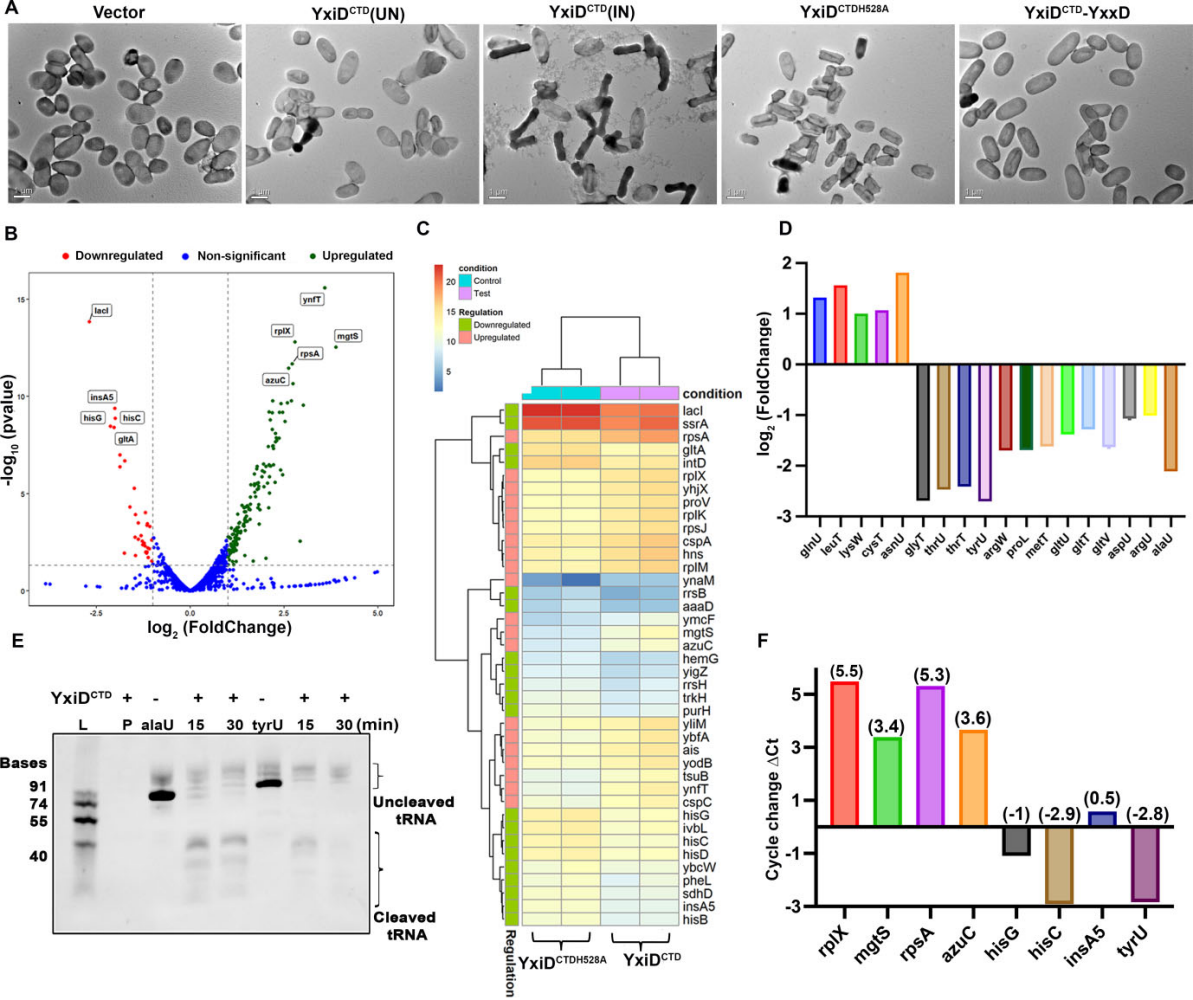
Effects of YxiD^CTD^ toxin at a cellular and molecular level. **(A)** TEM images of *E. coli* cells treated with toxin for 3 h showing structural alterations as compared to the mutant YxiD^CTDH528A^. **(B)** Volcano plot representing the differential gene expression analysis, highlighting log2-fold change based on differential gene expression analysis in YxiD^CTD^ compared to YxiD^CTDH528A^. Each point in the scatter plot represents a gene; the *x*-axis represents the log2-fold change, indicating the gene expression differences between the wildtype and the active site mutant, while the *y*-axis represents the statistical significance, typically shown as the negative logarithm of the adjusted *P*-value, highlighting the confidence level of the observed changes. Significantly upregulated and downregulated genes are shown as green and red points, respectively. **(C)** Heatmap showing top 20 differentially expressed genes in active toxin relative to the mutant, with color intensity representing expression levels. **(D)** Bar graph depicting the fold change in tRNAs upon toxin overexpression. **(E)** tRNase activity of YxiD^CTD^ toxin. 1 µg of AlaU (tRNA-AlaU^UGC^) and TyrU (tRNA-TyrU^GUA^) were incubated with 10 μM of YxiD^CTD^ protein for 15 and 30 min at 37°C. **(F)** RT-qPCR validation of RNA-seq results, validating the expression pattern of key upregulated and downregulated differentially expressed genes (DEGs).

To further probe changes in the cellular morphology and understand the mechanism of growth inhibition upon toxin overexpression, we performed RNA-Seq and transmission electron microscopy (TEM) experiments. Induced and uninduced cells, harboring toxin, and vector as a control, post 3 h of incubation, were visualized under TEM. The micrographs revealed that the toxin expression led to gross morphological changes, membrane disruption, and altered cell shape compared to uninduced cells. Interestingly, co-expression of the toxin, YxiD^CTD^, with YxxD neutralized the toxic effects, resulting in morphologically healthier cells. The loss of toxicity was also observed in YxiD^CTDH528A^, suggesting that His528 is important for the toxicity (Fig. 4A).

RNA-Seq studies were performed to analyze the transcriptomic changes upon toxin overexpression compared to its mutant variant. The data revealed significant alterations in gene expression profiles as shown in the volcano plot and heatmap (Fig. 4B and C). Around 187 genes showed differential expression, where 153 genes were upregulated, and 34 genes were downregulated. The volcano plot further illustrates these changes, with genes exhibiting statistically significant differential expression (adjusted *P*-value < 0.05) plotted based on their log_2_-fold change (Fig. 4B). Active toxin expression resulted in downregulation of genes involved in amino acid (notably histidine) biosynthesis pathways and upregulation of ribonucleoprotein complex, RNA-binding genes (Supplementary Figure S4 & S5) (Fig. 4C). A total of 153 genes were identified as upregulated, of which 57 are classified as essential. These primarily included genes involved in ribosome assembly and biogenesis, ribonucleoprotein complex biogenesis, RNA binding, and cytoplasmic translation, indicating an enhanced demand for protein synthesis machinery, possibly as a compensatory response to the cellular stress (Supplementary Table 3) [54, 67]. Interestingly, 3 out of the 34 downregulated genes, i.e. *coaA, hemG*, and *murI*, are classified as essential. Notably, the *murI* gene is essential for biosynthesis of D-Glutamate, a crucial component of bacterial peptidoglycan that contributes to the rigidity and structural integrity of the bacterial cell wall. This finding correlates with our TEM observations, which revealed significant membrane disruption in toxin-treated bacterial cells. The *coaA* gene encodes pantothenate kinase, a key enzyme in CoenzymeA biosynthesis, precursor for fatty acid synthesis and acetylation reactions. So, it indirectly supports cell wall biosynthesis by enabling lipid metabolism and the production of precursors essential for cell wall component assembly. The *hemG* gene indirectly supports cell wall biosynthesis by enabling heme production, which fuels respiration and redox reactions essential for lipid synthesis (Supplementary Table 4) [54, 67].

We examined tRNA levels in response to toxin overexpression in the cells and observed significant downregulation of several tRNA transcripts in cells expressing the active toxin, suggesting that YxiD^CTD^ may possess tRNase activity (Fig. 4D). The reduction in tRNAs could either be due to tRNase activity or could also be an indirect effect of altered expression of other genes. So, taking cues from the structural analysis and RNA-Seq data, we first performed an RNA cleavage assay using phage RNA as a substrate. We observed degradation of MS2 RNA in the presence of active toxin, indicating that YxiD^CTD^ is an RNase (Supplementary Fig. S6). To further check the specificity of this toxin, we selected *E. coli* tRNAs that were downregulated in the presence of toxin overexpression. To further investigate whether the downregulated tRNAs are direct substrates of YxiD^CTD^, *in vitro* cleavage assays were performed which confirmed that tRNA-AlaU^UGC^ and tRNA-TyrU^GUA^ were efficiently cleaved by YxiD^CTD^ (Fig. 4E). In addition to AlaU and TyrU, we observed cleavage of tRNA-GlyT^UCC^, tRNA-ThrU^UGU^, and tRNA-ThrT^GGU^ (Supplementary Fig. S7), though the efficiency was comparatively lower. Additionally, we investigated whether YxiD^CTD^ could target tRNAs from other bacterial species as well. As our lab works on several *Mtb* toxins and tRNAs, we tested YxiD^CTD^ activity against *Mtb* tRNA as well. Notably, tRNA-ValU^GAC^ from *Mtb* exhibited cleavage when incubated with YxiD^CTD^, suggesting a broad substrate range across different species, potentially contributing to microbial competition. This suggests that YxiD^CTD^ cleaves multiple tRNAs, having broad substrate specificity. Together, these results establish YxiD^CTD^ as a tRNase, supporting its role as a toxin that interferes with translation by directly degrading tRNA molecules.

To assess metal ion dependency, we performed cleavage reactions in the presence of both Mg^2+^ and Mn^2+^ ions. The toxin exhibited site-specific cleavage in the presence of Mg^2+^, whereas Mn^2+^ accelerated RNA degradation, leading to a more promiscuous cleavage pattern. These findings suggest that YxiD^CTD^ functions as a metal-dependent tRNase, with Mg^2+^ enforcing controlled specific cleavage and Mn^2+^ promoting rapid hydrolysis (Supplementary Fig. S8A). This observation is in agreement with other studies on RNases where activity is slow in the presence of Mg^2+^ while cleavage is fast and non-specific in the presence of Mn^2+^ [52, 68, 69].

To investigate the role of the anticodon loop in substrate recognition, we generated anticodon loop mutants for both *Mtb* tRNA-ValU^GAC^ and *E. coli* tRNA-TyrU^GUA^. Urea-PAGE analysis revealed that substitution of the native anticodon loop sequence eliminated cleavage of *Mtb* tRNA-ValU^GAC^, in contrast to *E. coli* tRNA, which remained susceptible to YxiD^CTD^-mediated cleavage (Supplementary Fig. S8B and C). Notably, YxiD^CTD^ did not cleave the upregulated tRNA-AsnU^GUU^ and tRNA-GlnU^UUG^, further indicating that cleavage is determined by sequence and/or structural determinants, probably within the anticodon loop in *Mtb* tRNA. These findings suggest that the anticodon loop serves as a critical determinant for YxiD^CTD^ recognition of *Mtb* tRNA-ValU^GAC^, whereas *E. coli* tRNA cleavage occurs independently of the anticodon loop sequence.

To examine the role of the cognate immunity protein YxxD in neutralizing YxiD^CTD^ toxicity, we performed *in vitro* cleavage assays with *Mtb* tRNA-ValU^GAC^ as a substrate in the presence of increasing concentrations of YxxD. The results showed that YxxD effectively inhibited YxiD^CTD^-mediated cleavage of tRNA-ValU^GAC^ in a concentration-dependent manner. At lower substoichiometric concentrations of YxxD, cleavage of the tRNA was still evident, whereas at 1:1 stoichiometric concentration or higher, no cleavage was observed (Supplementary Fig. S9). These findings demonstrate that YxxD neutralizes YxiD toxicity by directly inhibiting its tRNA cleavage activity.

RT-qPCR was performed to validate the differential gene expression observed in RNA-seq data. Specific primers targeting the key genes identified as significantly upregulated and downregulated were designed. The RT-qPCR data showed a strong correlation (seven out of eight genes) with the RNA-Seq data. Gene expression levels were normalized to the housekeeping gene *rpoD* to ensure reliable quantification, and relative expression changes were calculated using the 2^–ΔΔCt^ method (Fig. 4F). The data highlights the toxin’s ability to exert broad regulatory effects and modulate cellular response at the transcriptomic level. Taken together, YxiD^CTD^ is a potent metal ion-dependent RNase capable of cleaving multiple tRNAs, leading to tRNA depletion, hence disrupting protein synthesis and inducing cell death.

### Mutation of the active site and probable substrate-binding residues leads to abrogation of tRNase activity

**Figure 5.**
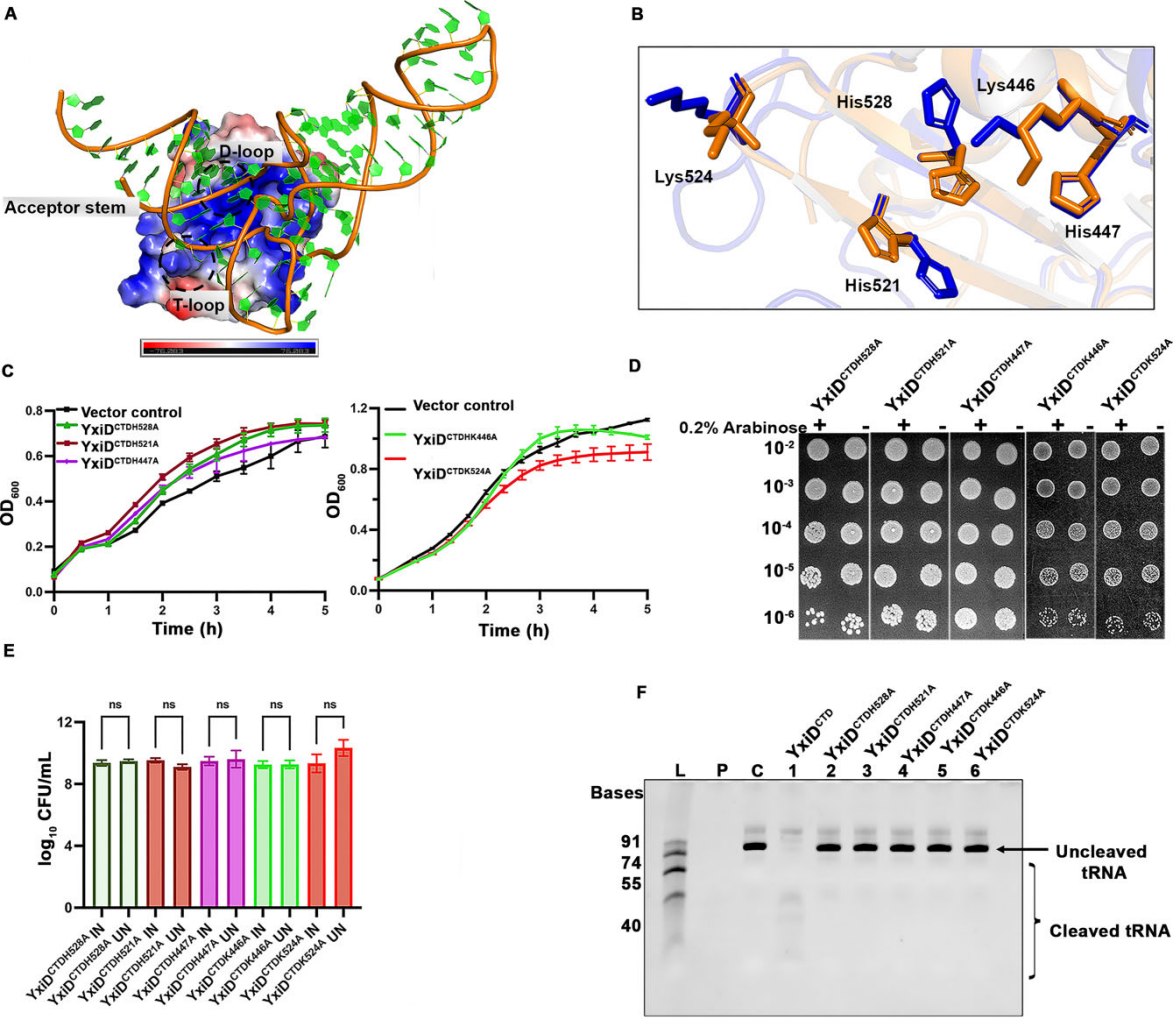
Mutation of the active site and predicted substrate-binding residues results in loss of tRNase activity. **(A)** Docking model of YxiD^CTD^ with *E. coli* tRNA-AlaU^UGC^ (structure predicted using AlphaFold 3) [71] using HDOCK server [70]. Electrostatic potential representation of YxiD^CTD^ is shown bound with tRNA-AlaU^UGC^, representing that tRNA binds to the positively charged active site, with lysine residues playing a central role in substrate recognition and binding. **(B)** The active site residues include three histidine residues, His528, His521, and His447, superposed with NMafB2-CT2(YxiD^CTD^ residues shown in blue color, NMafB2-CT2 residues shown in orange). **(C)** Growth curve analysis depicting the growth in the mutants as compared to the active toxin. **(D)** Dilution spotting analysis showing the active site mutants' growth inhibition relative to the toxin. Serial dilutions (10^-2^ to 10^-6^) are indicated on the Y-axis. **(E)** CFU Count represents the quantification of viable cells in the mutants with reference to the wildtype (IN represents induced samples and UN represents uninduced samples). Significance is indicated as ns = not significant (*P* > 0.05). **(F)** 10% Urea-PAGE gel showing RNA cleavage activity of purified YxiD^CTD^ mutants with tRNA-AlaU^UGC^. Abbreviations: L, Ladder; P, protein alone; C, negative control for tRNA-AlaU^UGC^. Data is representative of three independent experiments (*n* = 3).

Not many structures of related proteins from *B. subtilis* are known, so the precise mode by which the receptor interacts with its nucleic acid substrate remains poorly understood. To gain insights into the molecular basis of substrate recognition, we performed comparative molecular docking analysis of YxiD^CTD^ with two distinct tRNA substrates: tRNA-AlaU^UGC^ and tRNA-ValU^GAC^. Protein-tRNA substrate docking was carried out using the HDOCK server [70]. HDOCK integrates a fast Fourier transform (FFT)-based global docking algorithm along with an improved shape-based scoring function, allowing us to sample a wide range of putative binding modes. In the case of YxiD^CTD^-tRNA-AlaU^UGC^ complex, 100 docking models were generated, from which the highest-ranking models (with docking scores ranging from −269.49 to −240.19) were manually inspected. We selected a model with a confidence score of 0.89, where the substrate was favorably oriented at the active site of the toxin (Fig. 5A). Similar interactions were observed in the analysis of YxiD^CTD^-tRNA-ValU^GAC^ docking model, where conserved positively charged residues contributed to the stabilization of the RNA–protein interface (Supplementary Fig. S10). The recurrence of lysine-mediated contacts across both tRNA complexes suggests a conserved mechanism of substrate recognition by YxiD^CTD^, potentially driven by electrostatic complementarity and specific recognition of conserved structural motifs within the tRNA. Our detailed analysis of the docking interface revealed several key receptor residues that are likely crucial for substrate binding. More specifically, Lys446 and Lys524 interact closely with ligand residues. These interactions indicate that these lysine residues may play a pivotal role in substrate recognition and complex stabilization.

The binding cleft of YxiD^CTD^ is characterized by three evolutionary conserved histidine residues, His528, His521, and His447, which together form the active site. In addition to these, adjacent lysine residues Lys446 and Lys524 contribute to the formation of a positively charged surface that facilitates substrate binding, likely through electrostatic interactions with the negatively charged phosphate backbone of tRNA (Fig. 5B). To evaluate the role of these conserved histidine and lysine residues mediating toxicity/substrate binding, site-directed mutagenesis was carried out. Substitution of these residues with alanine resulted in a restoration of bacterial growth compared to cells expressing the wild-type toxin, suggesting that these residues are essential for the toxicity (Fig. 5C). Further dilution spotting experiments also supported this data, where the mutants showed reduced growth inhibition as compared to the wild-type toxin, suggesting impaired toxicity (Fig. 5D). Additionally, CFU count experiments demonstrated a marked increase in the viable cell count in the mutant strains as compared to the toxin-expressing cells (Fig. 5E).

To check the activity of these mutants, we performed a tRNA cleavage assay. As expected, all three active site mutants, YxiD^CTDH528A^, YxiD^CTDH521A^, and YxiD^CTDH447A^, resulted in a loss of activity. Interestingly, YxiD^CTDK446A^ and YxiD^CTDK524A^ lysine mutants also resulted in loss of activity, indicating the functional importance of these residues in substrate binding (Fig. 5F, Supplementary Fig. S8D). Taken together, these results indicate that the targeted active site residues, along with Lys446 and Lys524 probably involved in substrate binding, are critical for toxin-mediated growth inhibition, and their mutation significantly diminishes the toxin’s tRNase activity. In summary, our docking study not only provides a plausible model for the receptor-ligand interaction but also highlights specific residues that could be targeted in further biochemical or structural studies to elucidate the mechanism of substrate recognition.

## Discussion

PTS found across both Gram-positive and Gram-negative species serve as molecular weapons in bacterial competition, kin-selection, and niche adaptation [5]. While Gram-negative bacteria predominantly deploy contact-dependent growth inhibition (CDI) systems (e.g. CdiA-CdiI), Gram-positive species utilize LXG-domain toxins, typically secreted through the Type VII secretion system (T7SS). The RNase activity of polymorphic toxins often targets conserved RNA species such as tRNAs or rRNAs, highlighting an evolutionary adaptation to disrupt protein synthesis in competing bacteria [1, 11, 32]. In Gram-negative bacteria, such as *E. coli* and *Burkholderia pseudomallei*, tRNA-cleaving toxins are well-documented within CDI systems [72, 7]. For instance, *E. coli* CdiA-CT targets tRNA-Ala and tRNA-His, leading to translational arrest and cell death [1]. These RNase CDI toxins often harbor the BECR fold, a structural motif also conserved in some Gram-positive RNase toxins reflecting conserved mechanisms of RNase activity [32, 33]. While BECR fold toxins are structurally conserved, their immunity proteins are highly divergent. The study by Gucinski *et al.* (2019) reported that the *C*-terminal toxin domains of CdiA^EC3006^ and CdiA^Kp342^ from *E. coli* and *Klebsiella pneumoniae*, respectively, despite being derived from different bacterial species, share strong structural similarity and exhibit specific tRNase activity against the acceptor stem of tRNA-Ile^GAU^. These toxins independently evolved the BECR fold, converging on similar structures and substrate specificities despite mechanistic and immunity protein differences [62]. This highlights evolutionary pressure to target conserved tRNA substrates in interbacterial competition [2, 62]. The conservation of the BECR fold across diverse bacterial lineages highlights its structural efficiency and evolutionary advantage as a catalytic core in RNA-targeting toxins. Its widespread presence in both Gram-negative and Gram-positive PTS reflects convergent selection for an adaptable RNase fold capable of mediating interbacterial competition [39, 62, 73]. On the other hand, uropathogenic *E. coli* 536 adopts a different structural fold and mechanism of tRNA cleavage [72].

**Figure 6.**
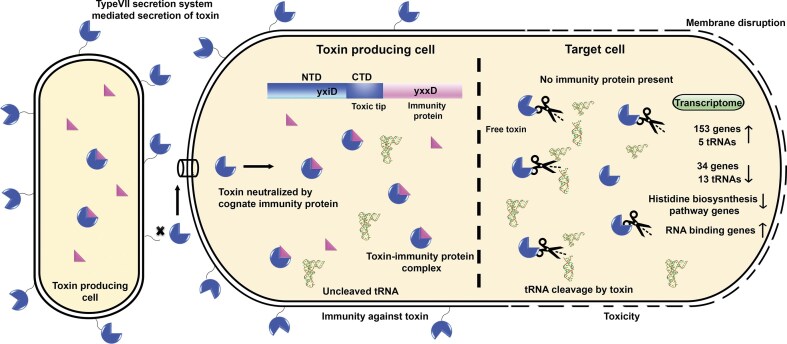
Proposed model illustrating YxiD^CTD^ toxin activity and neutralization by its cognate immunity protein, YxxD, in *B. subtilis* 6633. A toxin-producing (self) cell translocates YxiD polymorphic toxin via the Type VII secretion system (T7SS) to the cell surface. YxiD consists of a conserved *N*-terminal LXG domain (acts as an export signal to target toxin to the secretion apparatus) and a variable *C*-terminal toxic domain with tRNase activity. The cognate immunity protein (YxxD) co-expressed in the producer cell binds and neutralizes the toxin by sterically occluding its RNA-binding site. In contrast, in a “non-self” competing cell lacking the YxxD immunity protein, internalizes the active toxin, causing targeted tRNA cleavage, translational arrest, and transcriptional reprogramming leading to cell death. This provides a competitive advantage to the cells harboring the YxiD-YxxD PTS module over those that lack this module.

The similarity of *B. subtilis* toxins to these CDI effectors underlines evolutionary convergence across phyla. Sequence alignment using Clustal Omega [74] revealed that the *N*-terminal sequence of YxiD from *B. subtilis* 168 and *B. subtilis* 6633 shares 97% sequence identity over 362 residues, while the *C*-terminal domains share only 23% significant identity. This indicates a strong conservation of the *N*-terminal domain (involved in protein transport) and variability of the *C*-terminal domain (toxin activity), a characteristic of polymorphic toxins [18, 31]. Holberger *et al. *(2011) demonstrated that the *C*-terminal domain of YxiD (YxiD-CT) from *B. subtilis* 168 possesses ribonuclease activity targeting rRNA [32]. Our findings demonstrate that the YxiD^CTD^ from *B. subtilis* 6633 exhibits metal ion-dependent tRNase activity (Fig. 6), reiterating functional diversification of the toxins across strains.

*B. subtilis* 168 encodes another polymorphic toxin, WapA, which, like YxiD, features a conserved *N*-terminal domain and a variable *C*-terminal toxin domain. However, unlike YxiD, WapA is transported across the membrane through the SecA-dependent secretion pathway and is anchored to the cell wall, to facilitate contact-dependent growth inhibition (CDI) [17]. Interestingly, WapA toxin also harbors RNase activity and the toxic effects of wapA are observed under planktonic growth conditions but are absent under biofilm conditions [33]. So, it appears that bacteria utilize different toxins for different growth and environmental conditions. RNA-Seq and TEM analysis demonstrated that YxiD^CTD^ expression leads to broad transcriptional dysregulation, particularly among pathways related to amino acid metabolism, ribosome biogenesis, and RNA–protein complex assembly, and significant cellular damage, including membrane disruption resulting in toxicity.

Despite the diversity of toxic domains, there is notable structural conservation in the delivery mechanisms of PTS across bacterial species. The conserved *N*-terminal domains, such as RHS, or LXG motifs in Gram-negative and Gram-positive bacteria, respectively, serve as stable scaffolds, while the *C*-terminal modules undergo diversification [2, 3, 5]. This architecture supports PTS to adapt to diverse ecological niches and interbacterial warfare. This study again exemplifies this basic architecture, where the *N*-terminal domain in YxiD toxins in two closely related *B. subtilis* strains is highly conserved while the *C*-terminal domain shows poor sequence conservation and differences in molecular targets. These PTS systems often target closely related bacterial strains or species. In our study, we observed *in vitro* cleavage of several tRNAs from heterologous hosts, *E. coli* and *Mtb*, by YxiD, suggesting a broad substrate specificity. However, toxicity in the target cells is also determined by the presence of receptors that aid the uptake/import of these toxins. These import mechanisms determine strain/species specificity [62, 75–78]. Previous studies have shown that bacteria respond to RNase toxicity by expressing RNA chaperones (e.g. Hfq, ProQ) or RNA helicases that promote repair or degradation of damaged RNA [79]. Our RNA-Seq data suggested upregulation of several RNA-binding genes, suggesting that these proteins may also play a role in overcoming toxicity caused by the presence or expression of RNase toxin. However, detailed studies are required to investigate the role of these RNA-binding proteins in overcoming RNase toxicity.

To summarize, we have solved the crystal structure of YxiD^CTDH528A^-YxxD complex and have established that it is a broad-spectrum tRNase. We used structural, biophysical, and biochemical approaches to characterize YxiD^CTD^-YxxD complex. While tRNA-targeting toxins have been characterized in other bacterial species, most exhibit species-restricted activity, with cleavage limited to closely related organisms. The ability of YxiD^CTD^ to cleave tRNA from phylogenetically diverse bacteria suggests a probable expanded functional role in bacterial interactions. The cross-species activity of YxiD^CTD^ and its role in interspecies competition that may contribute to broader ecological interactions warrant detailed investigations. Unlike previously characterized polymorphic toxins that primarily act within a narrow host range, YxiD^CTD^ demonstrates substrate flexibility in targeting tRNAs from phylogenetically distinct species. The differential cleavage patterns indicate that the toxin may recognize specific structural features unique to the tRNAs. This adaptability could be an evolutionary advantage, allowing the toxin to effectively inhibit protein synthesis across diverse bacterial species. We also show that YxiD^CTD^ recognition of *Mtb* tRNA-ValU^GAC^ is critically dependent on the anticodon loop sequence, whereas *E. coli* tRNA cleavage occurs probably through an alternative recognition pathway independent of the anticodon loop. Further structural and biochemical studies are warranted to elucidate the precise determinants of substrate recognition and cleavage. Understanding these mechanisms may offer insights into bacterial stress responses. Further investigation is required into YxiD’s physiological role within bacterial populations. Competition assays will help determine the ecological relevance of YxiD-mediated killing in native bacterial communities. Additionally, high-resolution structural studies with RNA substrates could provide deeper insights into the specificity determinants of this toxin’s activity.

## Supplementary Material

gkaf1321_Supplemental_File

## Data Availability

The crystal structure coordinates and structure factor file have been deposited in the RCSB Protein Data Bank under the PDB ID: 9V8I. RNA-Seq data have been deposited in the NCBI Sequence Read Archive (SRA) under the accession number PRJNA1257277.

## Notes

Present address- Verna and Marrs McLean Department of Biochemistry and Molecular Pharmacology at Baylor College of Medicine, Houston, TX 77030, United States

Present address- Department of Biotechnology, National Institute of Pharmaceutical Education & Research (NIPER), S.A.S. Nagar, Punjab 160062, India
