# Supplementary material for: The yxiD gene from Bacillus subtilis 6633 encodes a polymorphic toxin with tRNase activity and is neutralized by the cognate immunity protein YxxD
Source: Nucleic Acids Res. 2025 Dec 12;53(22):gkaf1321. doi: 10.1093/nar/gkaf1321 (PMC12700104; doi:10.1093/nar/gkaf1321)
Supplement: gkaf1321_Supplemental_File [file gkaf1321_supplemental_file.pdf]

Supplementary Data

The *yxiD* gene from *Bacillus subtilis* 6633 encodes a polymorphic toxin with tRNase activity and is neutralized by the cognate immunity protein YxxD

Rishita Rohilla[1,2], Soni Kaundal[1,3], Krishan Gopal Thakur[1,2,4*]

[1]Structural Biology Laboratory, CSIR-Institute of Microbial Technology, Chandigarh-160036, India
[2]Academy of Scientific and Innovative Research (AcSIR), Ghaziabad, India
[3]Present address- Verna and Marrs McLean Department of Biochemistry and Molecular Pharmacology at Baylor College of Medicine, Houston, Texas, USA
[4]Present address- Department of Biotechnology, National Institute of Pharmaceutical Education & Research (NIPER), S.A.S. Nagar-160062, Punjab, India
*Corresponding Author: krishang@imtech.res.in, krishang@niper.ac.in

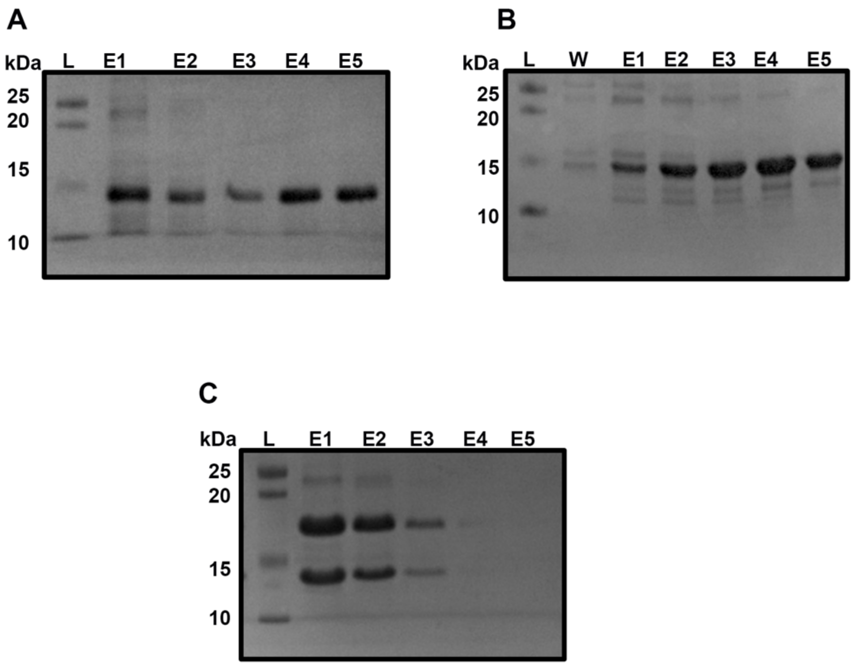

Supplementary Figure S1: SDS-PAGE analysis for expression and purification of **(A)** YxiD^{CTD}, **(B)** YxxD and **(C)** YxiD^{CTD}-YxxD complex**.** Protein samples were resolved on a 15 % SDS-PAGE gel (L, protein ladder; W, wash; E1-E5, elution fractions).

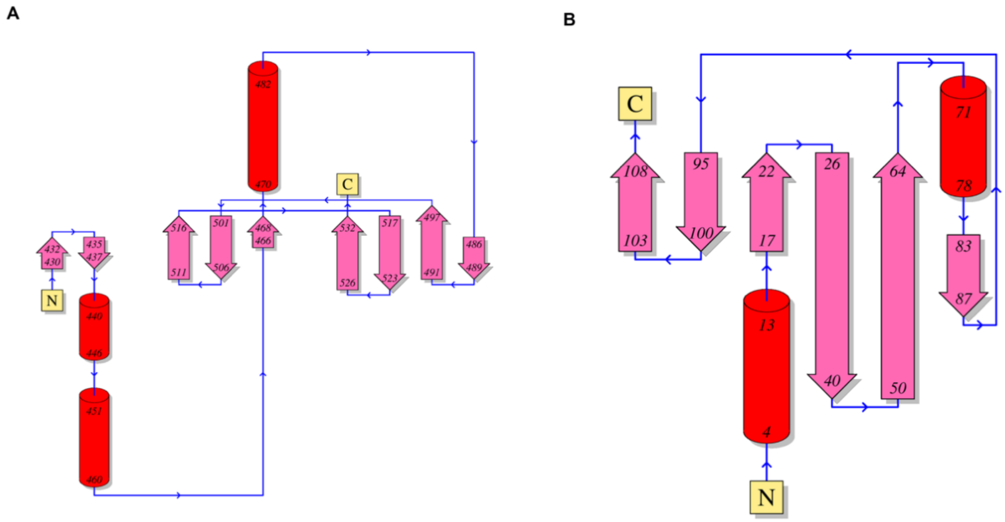

Supplementary Figure S2: Topology diagram of **(A)** YxiD^{CTD} **(B)** YxxD

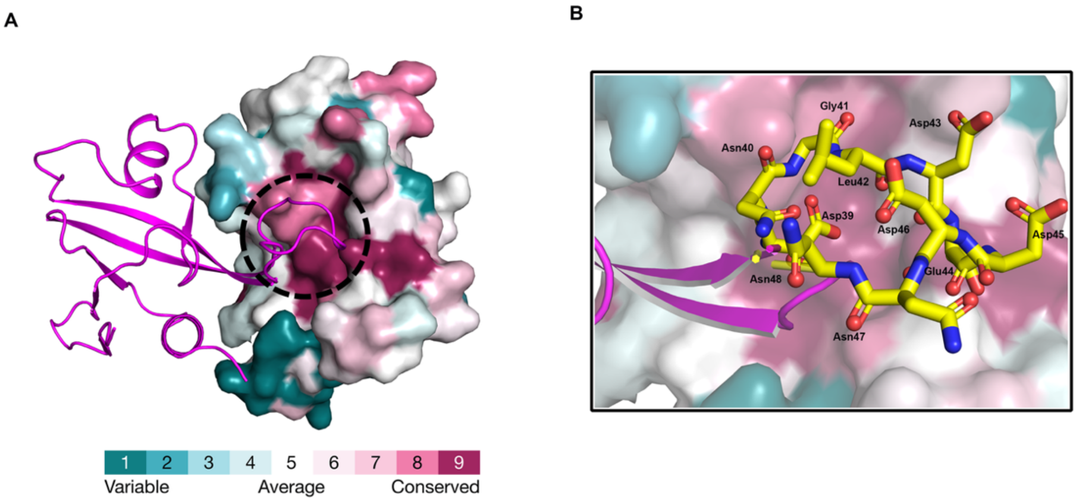

Supplementary Figure S3: Surface conservation analysis of YxiD^{CTD} toxin using Consurf Server. **(A)** YxiD^{CTD} toxin is shown in surface representation showing that the active site pocket is evolutionary conserved and YxxD is shown in cartoon schematic (magenta). The dotted circle represents the negatively charged residues blocking the toxin interface. **(B)** The loop region extending from β2- β3 is shown in stick representation (yellow), highlighting the residues involved in toxin neutralization.

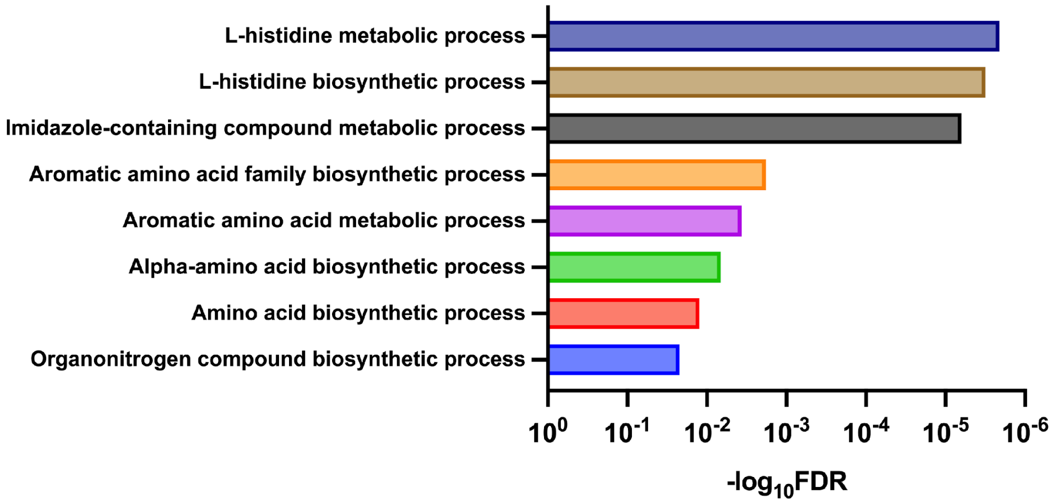

Supplementary Figure S4: Metabolic processes downregulated when YxiD^{CTD} is overexpressed. The figure shows top enriched biological processes for downregulated genes (based on p-value).

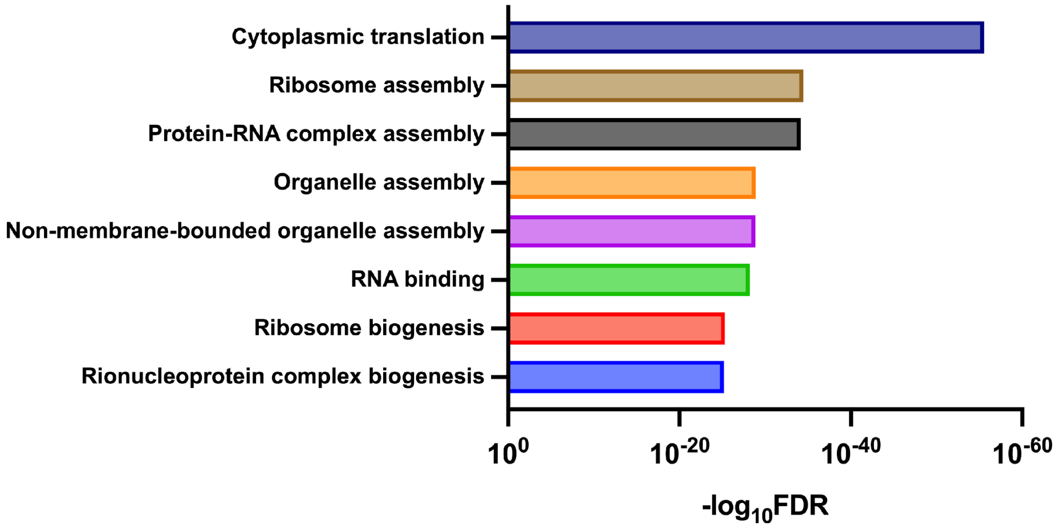

Supplementary Figure S5: Metabolic processes upregulated when YxiD^{CTD} is overexpressed. The figure shows top enriched biological processes for upregulated genes (based on p-value).

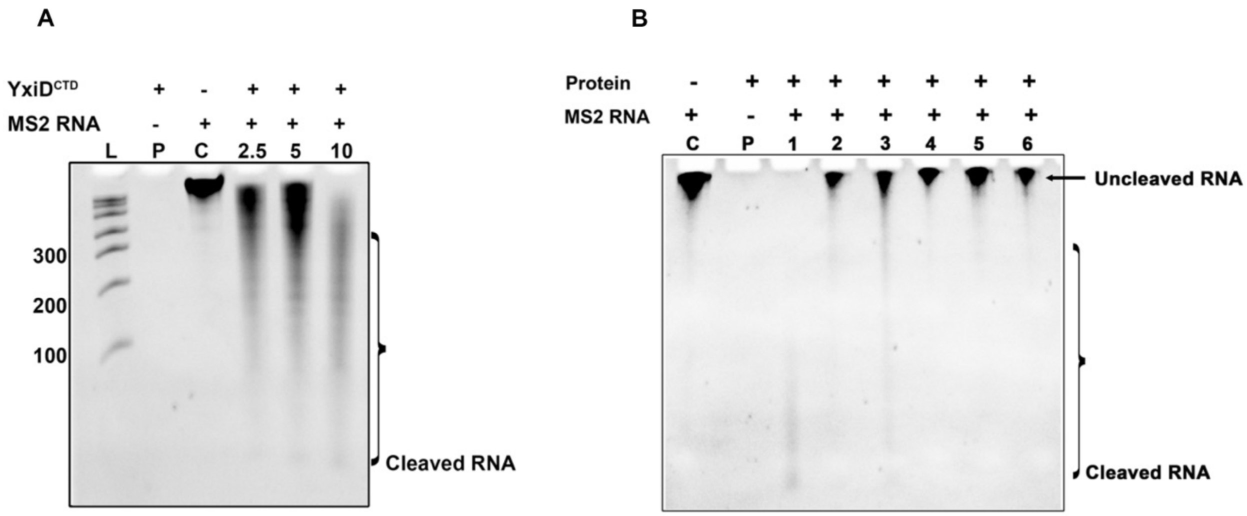

Supplementary Figure S6: RNase activity of YxiD^{CTD} toxin. **(A)** RNA Cleavage assay demonstrating the RNase activity of YxiD^{CTD}. 6% Urea-PAGE gel showing RNA cleavage activity of purified YxiD^{CTD} with MS2 RNA. 1 µg of MS2 RNA was incubated with 2.5, 5 and 10 µM protein for 10 min at 37 °C. **(B)** YxiD^{CTD} mutants do not cleave RNA. 1, YxiD^{CTD}; 2, YxiD^{CTDH528A}; 3, YxiD^{CTDH521A}; 4, YxiD^{CTDH447A}; 5, YxiD^{CTDK446A} and 6, YxiD^{CTDK524A}. (L - Ladder, P - protein alone and C - MS2 RNA alone).

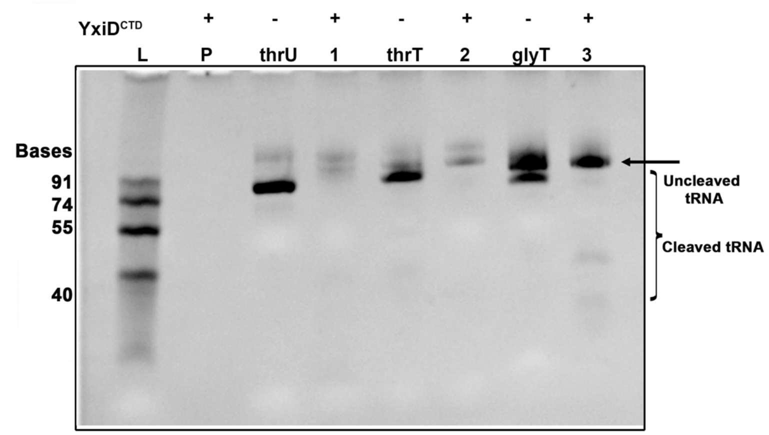

Supplementary Figure S7: Urea-PAGE Analysis of tRNA cleavage for transcripts downregulated in RNA-Seq data. 1, tRNA-thrU^{UGU}; 2, tRNA-thrT^{GGU}; 3, tRNA-glyT^{UCC} incubated with 10 μM of YxiD^{CTD} protein for 15 min at 37 °C.

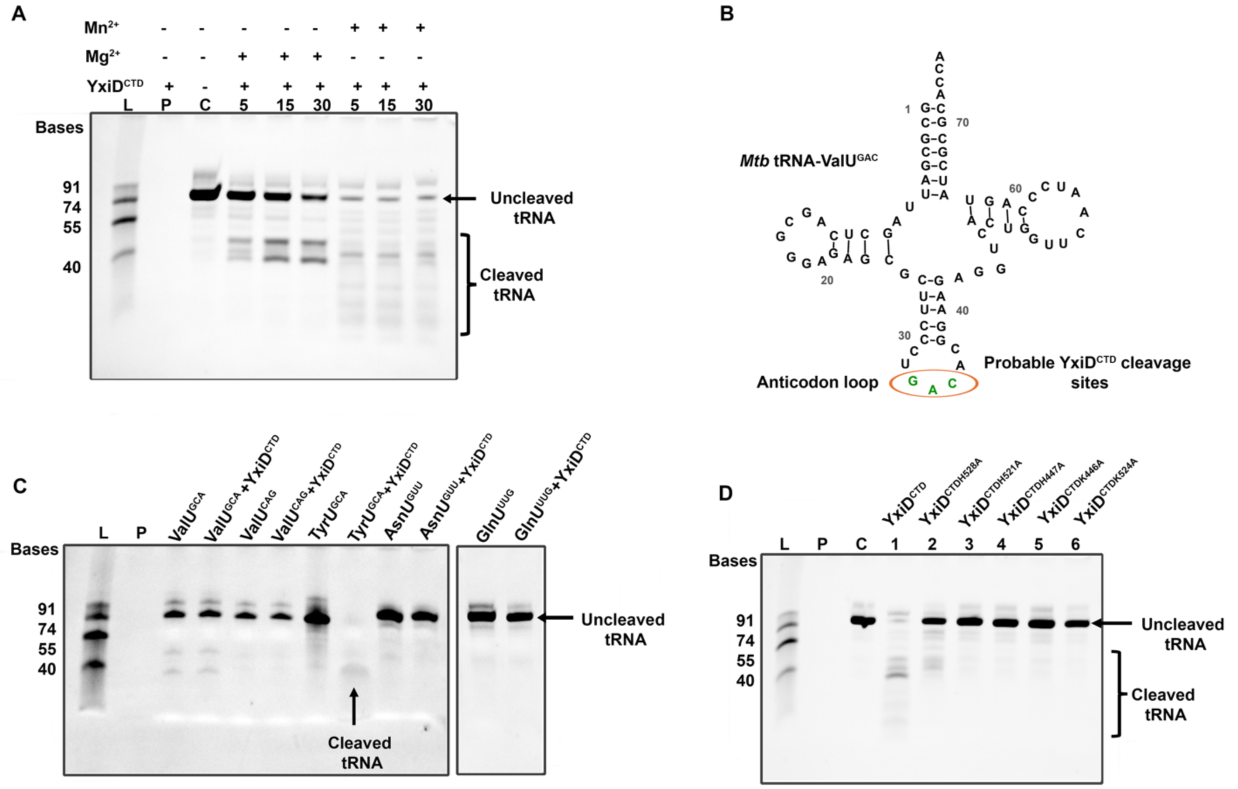

Supplementary Figure S8: *In vitro* cleavage assay showing cleavage of *Mtb* tRNA-ValU$^{GAC.}$ **(A)** 10% Urea-PAGE gel showing cleavage of tRNA-ValU^{GAC} in the presence of metal ions. **(B)** Schematic representation of tRNA-ValU^{GAC} showing probable YxiD^{CTD} cleavage sites. **(C)** 10% Urea-PAGE gel showing cleavage of anticodon loop mutants tRNA-ValU^{CAG}, tRNA-ValU^{GCA}, tRNA-tyrU^{GCA}. Mutation of the anticodon loop abolished cleavage of *Mtb* tRNA, while *E. coli* tRNA remained susceptible. No cleavage was observed in representative upregulated tRNAs, tRNA-AsnU^{GUU} and tRNA-GlnU^{UUG} **(D)** 10% Urea-PAGE gel analysis of tRNA cleavage assay of tRNA-ValU^{GAC} with YxiD^{CTD} mutants. No tRNA cleavage activity was observed in these mutants.

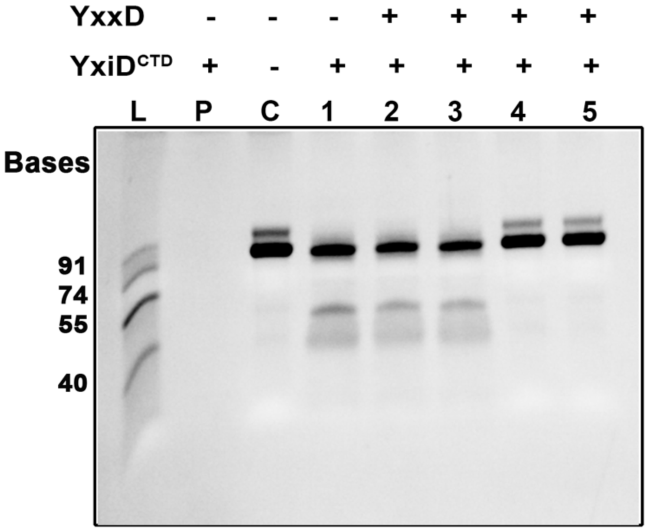

Supplementary Figure S9: YxxD neutralizes YxiD toxicity by inhibiting toxin activity. *In vitro* cleavage assay showing inhibition of *Mtb* tRNA-ValU^{GAC} cleavage in the presence of YxxD. 500ng of *Mtb* tRNA-ValU^{GAC} was incubated with 10 µM YxiD^{CTD} in the presence of increasing concentrations of YxxD (1, 5, 10 and 20 µM). Cleavage of the tRNA was observed at lower concentrations of YxxD, whereas no cleavage was detected at 10 µM and 20 µM YxxD.

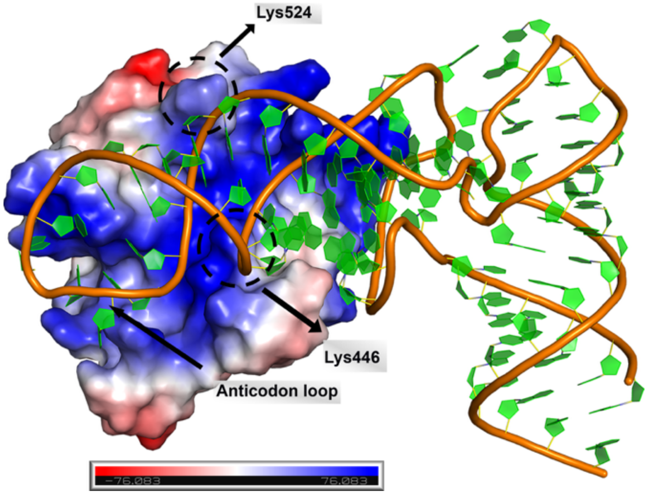

Supplementary Figure S10: Docking model of YxiD^{CTD} with tRNA- ValU^{GAC} (structure predicted using AlphaFold). Electrostatic potential representation of YxiD^{CTD} shown bound with tRNA-ValU^{GAC} representing that tRNA binds to the positively charged active site of the toxin.

Supplementary Table 1: A list of constructs and primers made during the study.

S. No.	Primer Name	Nucleotide Sequence
1	YxiDCTD-6633-F408_BamHI	GCGGATCCAGTTGCTAAGGATACGGGTA ATGGTGTAC
2	YxiDCTD-6633-R_EcoRI	GCGAATTCTTATGGCCTAGATGGTACTAT ATGCGC
3	YxxD-6633-F_NdeI	GCAGCATATGGGCTATGATTCTTTAATTGAAA CTTTAATA
4	YxxD-6633-R_XhoI	ACGCTCGAGTTAATTATCGCTTTTCCACA CTCTTTG
5	T7 Promoter	TAATACGACTCACTATAGGGGAATTGTG
6	T7 Terminator	CAAAAAACCCCTCAAGACCCG
7	YxiD_Mut_t_BamHI_Fwd	CATCACCACAGCCAGGATtCCATGAAGCC AGGTGC
8	pBAD Fwd	CGCAACTCTCTACTGTTTCTCCAT
9	pBAD Rev	GATTTAATCTGTATCAGGCTGAAAATCT
10	MCSI Rev	CGATTACTTTCTGTTCGACTTAAGCAT
11	MCSII Fwd	TTGTACACGGCCGCATAATC
12	YxiDCTD_K446A_Mut	AAAGTAAAGCCAGGTGCTCAAGAGGCACATA TTCCTAATACGCCAAATTACAAA
13	YxiDCTD_K524A_Mut	GACTACTAAAGGATTAATACATTATGGAGCA GACGGGGCGCATAT AGTACCAT
14	pBAD_YxiDCTD_NheI_Fwd	ACCATCATCACCACAGCCAGGCTAGCA AGCCAGGTGCTCAAGAGAAAC
15	pBAD_YxiDCTD_HindIII Rev	ATGAGTTTTTGTTCGGGCCCAAGCTTTT ATGGCCTAGATGGTACTATA

**16**	pBAD_YxiDCTDH528A_Rev_HindIII	GAGATGAGTTTTGTTCGGGCCCAAGCTTTTATGGCCTAGATGGTACTATGGCC
**17**	pDuet_YxxD_NheI_Fwd	CATCACCATCATCACCACAGCCAGGCTAGCATGGGCTATGATTCTTTAATTGAAACTTT
**18**	pDuet_YxxD_HindIII_Rev	CTTAAGCATTATGCGGCCGCAAGCTTTTAATTATCGCTTTTCCACACTCTTTG
**19**	tRNA_ValU_(GAC)_150bpUP_NheI Fwd	CTGCGCCACATGCATCGCTTTGAGGTGCTAGTGGCATCAA
**20**	tRNA_ValU_(GAC)_Fwd_T7 Promoter	TAATACGACTCACTATAGGGCGCGATTAGCTCAGCGG
**21**	tRNA_ValU_(GAC)_Rev	TGCGCGATACTGGGATTG
**22**	tRNA_ValU_(GAC)_150bpDownRev NheI	CTTGTCCAGAGCTCCCATATGGATTTAGCAAACTCCTCCG
**23**	pBAD_alaU_200bpUP_NheI_Fwd	CCATCATCACCACAGCCAGGCTAGCGATCACCTCCTTACCTTAAAGAAGCG
**24**	AlaU Fwd_T7 Promoter	TAATACGACTCACTATAGGGGGCTATAGCTCAGCTGGG
**25**	AlaU Rev	TGGTGGAGCTATGCGGGAT
**26**	pBAD alaU Rev HindIII	TGAGATGAGTTTTGTTCGGGCCCAAGCTTCACCGTGTACGCTTAGTCG
**27**	pBAD thrT Fwd NheI	ATCACCATCATCACCACAGCCAGGCTAGCCCAATTTCGGCCACGCGATG
**28**	ThrT Fwd	TAATACGACTCACTATAGGCTGATATAGCTCAGTTGGTAGAGC
**29**	ThrT Rev	TGGTGCTGATAGGCAGATTCGA
**30**	pBAD thrT Rev HindIII	TGAGATGAGTTTTGTTCGGGCCCAAGCTTGGTCAGCGTTGTTTTACCATGG
**31**	pBAD thrU Fwd NheI	CATCACCATCATCACCACAGCCAGGCTAGCCAGATATTCCATCTGATAAAAAGAATTATGGT
**32**	ThrU Fwd	TAATACGACTCACTATAGGCCGACTTAGCTCAGTAGGTAGAG
**33**	ThrU Rev	TGGTGCCGACTACCGGAA
**34**	pBAD thrU Rev HindIII	TGAGATGAGTTTTGTTCGGGCCCAAGCTTCTCGGCTACCTGATTTTCATTC

**35**	pBAD tyrU Fwd NheI	CATCACCATCATCACCACAGCCAGGCTAGCG CCCTCCGTTCGGCTGTT
**36**	TyrU Fwd	TAATACGACTCACTATAGGGTGGGGTTCCCG AGCG
**37**	TyrU Rev	TGGTGGTGGGGGAAGGATT
**38**	pBAD tyrU Rev HindIII	TGAGATGAGTTTTTGTTCGGGCCCAAGCTTAG CACATCTTGGAGCGGG
**39**	GlyT_qPCR_Fwd	TAATACGACTCACTATAGGCGGGCATCGTAT AATGGC
**40**	GlyT_qPCR_Rev	TGGAGCGGGCAGCGGGAA
**41**	MgtS_qPCR_Fwd	TGGCCGTACTGGGAATAATTT
**42**	MgtS_qPCR_Rev	CATCCCATTTGTGGCTGAAATACG
**43**	RplX_qPCR_Fwd	CAGGTTTCCAACGTAGCAATCTTC
**44**	RplX_qPCR_Rev	GAATCTAAAGCCTACACGGTCAGC
**45**	RpsA_qPCR_Fwd	CGCTAAAGGCGCAACCGTAGAA
**46**	RpsA_qPCR_Rev	CTTCAGAAGCACGCAGGTAAC
**47**	AzuC_qPCR_Fwd	CTGCGCAAAATCCTGAAAAGTATGT
**48**	AzuC_qPCR_Rev	GCCTGGCGGTACGTCT
**49**	InsA5_qPCR_Fwd	CTATCTCTGCTCTCACTGCCG
**50**	InsA5_qPCR_Rev	CCGGGTTGAGAAGCGGT
**51**	HisC_qPCR_Fwd	CTTTGTGGGATCAGGGCATTATC
**52**	HisC_qPCR_Rev	GCCGCTTAAAGAGGGTTGTTTATTC
**53**	GlyT_qPCR_Fwd	CGTATAATGGCTATTACCTCAGCCT
**54**	GlyT_qPCR_Rev	TGGAGCGGGCAGCG
**55**	HisG_qPCR_Fwd	CCGCTCTCCTTAAACGGTAAAC
**56**	HisG_qPCR_Rev	CGCTTGAGCAGGTGAGGATAAG
**57**	Duet 5’ Fwd	CGATCTCGATCCCGCGAAA
**58**	YxiDCTD_Mut_H521A	ATGCGCCCCGTCTTTTCCATAAGCTATTAATC CTTTAGTAGTCTCA
**59**	YxiDCTD_Mut_H447A	GTAATTTGGCGTATTAGGAATAGCTTTCTCTT GAGCACCTGGC

**60**	ValU_Mut_CAG_Fwd	GCGATTAGCTCAGCGGGAGAGCGCTTCCCTC AGACGGAAGAGGTCA
**61**	ValU_Mut_CAG_Rev	GATACTGGGATTGAACCAGTGACCTCTTCCGT CTGAGGGAAGCGCTCT
**62**	ValU_Mut_GCA_Fwd	GCGATTAGCTCAGCGGGAGAGCGCTTCCCTG CAACGGAAGAGGTCA
**63**	ValU_Mut_GCA_Rev	GATACTGGGATTGAACCAGTGACCTCTTCCGT TGCAGGGAAGCGCTCT
**64**	TyrU_mut_GCA_Fwd	TGGGGTTCCCGAGCGGCCAAAGGGAGCAGAC TGCAAATCTGCCGTCAC
**65**	TyrU_Mut_GCA_Rev	GTGGTGGGGGAAGGATTCGAACCTTCGAAGT CTGTGACGGCAGATTTGCAGTCTGCTCCCTT
**66**	AsnU 100up Fwd	AATGCAACAAACTGTTGATAGAAACG
**67**	AsnU 100Down Rev	GTTGAGGATATCAAGCGGCAG
**68**	AsnU Fwd	TAATACGACTCACTATAGTCCTCTGTAGTTCA GTCGGTAGAACG
**69**	AsnU Rev	TGGCTCCTCTGACTGGACTC
**70**	pET22b_YxiD-FL_Fwd	CCTCGCTGCCCAGCCGGCGATGGCCATGGGC TTGAAAACATTAGATGTCCACGCTC
**71**	pET22b_YxiD FL Rev	GTGGTGGTGGTGGTGCTCGAGTGGCCTAGAT GGTACTATATGCGC

Supplementary Table 2: OD_{600} measurements recorded at 40 min intervals corresponding to the growth curve shown in Figure 1C. Each data point is an average of three technical replicates.

**Time(h)**	**YxiD-FL**			**YxiD^{CTD}**			**YxiD^{CTD}-YxxD**			**YxxD**			**Control**		
0	0.0846	0.077	0.078	0.08	0.089	0.09	0.084	0.083	0.089	0.084	0.087	0.084	0.081	0.078	0.086
0.4	0.204	0.196	0.179	0.189	0.220	0.210	0.191	0.209	0.218	0.183	0.178	0.191	0.193	0.173	0.136
1.2	0.252	0.269	0.225	0.272	0.280	0.259	0.235	0.251	0.262	0.218	0.213	0.221	0.279	0.223	0.210
2	0.310	0.333	0.314	0.308	0.307	0.286	0.261	0.29	0.288	0.256	0.248	0.264	0.383	0.311	0.329
2.4	0.345	0.405	0.387	0.337	0.329	0.309	0.27	0.304	0.305	0.284	0.274	0.308	0.477	0.442	0.418
3.2	0.360	0.424	0.412	0.349	0.338	0.316	0.277	0.308	0.324	0.296	0.283	0.333	0.526	0.54	0.528
4	0.374	0.444	0.417	0.352	0.34	0.322	0.285	0.321	0.351	0.304	0.309	0.367	0.559	0.608	0.608
4.4	0.395	0.467	0.424	0.363	0.351	0.334	0.305	0.349	0.369	0.335	0.353	0.386	0.594	0.653	0.666
5.2	0.400	0.463	0.425	0.366	0.352	0.341	0.364	0.403	0.396	0.383	0.431	0.433	0.617	0.711	0.693
6	0.412	0.47	0.439	0.372	0.373	0.358	0.442	0.467	0.419	0.542	0.506	0.468	0.660	0.75	0.758
6.4	0.419	0.478	0.451	0.381	0.393	0.382	0.542	0.539	0.459	0.607	0.577	0.515	0.676	0.779	0.769
7	0.425	0.488	0.471	0.398	0.431	0.417	0.604	0.58	0.510	0.656	0.622	0.579	0.703	0.800	0.8
7.4	0.436	0.496	0.486	0.425	0.464	0.446	0.648	0.612	0.563	0.693	0.657	0.642	0.751	0.825	0.801
8.2	0.446	0.495	0.503	0.464	0.491	0.470	0.698	0.649	0.606	0.730	0.688	0.678	0.779	0.835	0.824
9	0.456	0.517	0.529	0.497	0.521	0.488	0.725	0.694	0.649	0.754	0.713	0.71	0.821	0.855	0.847
9.4	0.465	0.532	0.539	0.517	0.534	0.502	0.77	0.724	0.705	0.774	0.748	0.752	0.845	0.890	0.857
10.2	0.476	0.546	0.552	0.541	0.556	0.511	0.798	0.748	0.754	0.798	0.777	0.775	0.864	0.903	0.906

Supplementary Table 3: List of essential genes upregulated upon overexpression of YxiD^{CTD} toxin in *E. coli.*

S. No.	Gene	Gene symbol	Function	log_2FoldChange
**1.**	**b3309**	rplX	initiates ribosome assembly	2.78
**2.**	**b0911**	rpsA	RNA chaperone	2.70
**3.**	**b3983**	rplK	part of ribosome stalk	2.69
**4.**	**b3231**	rplM	early assembly protein of 50S ribosomal subunit	2.37
**5.**	**b3321**	rpsJ	involved in binding tRNA to the ribosome and plays a role in transcription antitermination by interacting with NusB	2.29
**6.**	**b3984**	rplA	binds near the 3'-end of 23S rRNA, implicated in tRNA translocation during protein synthesis.	2.21
**7.**	**b3986**	rplL	ensures accurate translation by binding to ribosomal stalk	2.20
**8.**	**b3319**	rplD	ribosome assembly	2.19
**9.**	**b3985**	rplJ	critical for translation elongation by interacting with elongation factors	2.19
**10.**	**b3306**	rpsH	binds to 16S rRNA, playing a role in the assembly of the 30S ribosomal subunit	2.18
**11.**	**b2185**	rplY	binds to 5S rRNA, role in ribosome assembly and function.	2.18
**12.**	**b3318**	rplW	binds 23S rRNA	2.13
**13.**	**b3320**	rplC	assembly initiator protein, it binds directly near the 3'-end of the 23S rRNA, nucleating assembly of the 50S subunit	2.10
**14.**	**b3165**	rpsO	primary rRNA binding protein that binds directly to 16S rRNA	2.05
**15.**	**b3310**	rplN	plays a role in subunit association	2.01
**16**	**b3340**	fusA	facilitates the movement of tRNA and mRNA	1.96
**17.**	**b3342**	rpsL	translational accuracy	1.95
**18.**	**b2609**	rpsP	cytoplasmic translation	1.95
**19.**	**b3307**	rpsN	contributes to the structural stability of the ribosome	1.91
**20.**	**b2608**	rimM	involved in the maturation of the 30S ribosomal subunit	1.90
**21.**	**b3316**	rpsS	contributes to the structure of the 30S ribosomal subunit	1.82
**22.**	**b4200**	rpsF	stabilize the platform of the 30S subunit	1.81
**23.**	**b3637**	rpmB	ribosome assembly	1.81

**24.**	**b3296**	rpsD	primary rRNA binding proteins; nucleates assembly of the body of the 30S subunit	1.78
**25.**	**b4202**	rpsR	helps stabilize the platform of the 30S subunit	1.77
**26.**	**b0169**	rpsB	required for ribosomal protein S1 to bind to the 30S subunit.	1.72
**27.**	**b4201**	priB	binds single-stranded DNA at the primosome assembly site (PAS	1.70
**28.**	**b3313**	rplP	binds directly to 23S rRNA and is located at the A site of the peptidyl transferase center.	1.70
**29.**	**b3315**	rplV	role in the assembly of the 50S ribosomal subunit	1.70
**30.**	**b3230**	rpsI	C-terminal tail plays a role in the affinity of the 30S P site for different tRNAs.	1.61
**31.**	**b3297**	rpsK	binds to 16S rRNA and is involved in the assembly of the 30S ribosomal subunit. It plays a role in maintaining the structure of the decoding center.	1.60
**32.**	**b3295**	rpoA	part of the core RNA polymerase enzyme responsible for transcription.	1.59
**33.**	**b2607**	trmD	catalyzes the methylation of guanosine at position 37 (m1G37) in tRNA, a modification crucial for maintaining the reading frame during protein synthesis	1.56
**34.**	**b4147**	efp	facilitates peptide bond formation during translation	1.52
**35.**	**b3317**	rplB	plays a role in peptidyl transferase activity.	1.51
**36.**	**b0023**	rpsT	binds directly to 16S rRNA and is involved in the assembly of the 30S ribosomal subunit.	1.47
**37.**	**b3314**	rpsC	binds to the lower part of the 30S subunit head and interacts with mRNA in the 70S ribosome	1.47
**38.**	**b3304**	rplR	mediates the attachment of the 5S rRNA subcomplex onto the large ribosomal subunit	1.47
**39.**	**b1089**	rpmF	involved in ribosome assembly and function	1.46
**40.**	**b3186**	rplU	binds to 23S rRNA and is involved in the assembly and structural stability of the 50S ribosomal subunit.	1.44
**41.**	**b3185**	rpmA	stabilizes the catalytic center of the ribosome.	1.34
**42.**	**b3461**	rpoH	master regulator of the heat shock regulon	1.33
**43.**	**b3311**	rpsQ	crucial role in the assembly of the 30S ribosomal subunit	1.29
**44.**	**b0170**	tsf	acts as a guanine nucleotide exchange factor for EF-Tu	1.25
**45.**	**b3298**	rpsM	involved in the assembly and function of the ribosome	1.25

**46.**	**b3341**	rpsG	initiates the assembly of the head domain of the 30S subunit	1.24
**47.**	**b3308**	rplE	binds and mediates the attachment of 5S rRNA into the large ribosomal subunit	1.24
**48.**	**b3339**	tufA	binds aminoacyl-tRNA and delivers it to the A-site of the ribosome during protein synthesis	1.22
**49.**	**b2614**	grpE	critical role in protein folding and the heat shock response	1.22
**50.**	**b3305**	rplF	role in the assembly of the 50S ribosomal subunit.	1.19
**51.**	**b0096**	lpxC	essential for the formation of lipopolysaccharides in the outer membrane of Gram-negative bacteria.	1.16
**52.**	**b3294**	rplQ	involved in the assembly and structural stability of the 50S ribosomal subunit	1.14
**53.**	**b3303**	rpsE	role in maintaining translational accuracy	1.13
**54.**	**b3783**	rho	essential ATP-dependent helicase that facilitates Rho-dependent transcription termination	1.11
**55.**	**b3302**	rpmD	role in ribosome assembly and function	1.11
**56.**	**b2530**	iscS	tRNA modification, and cofactor biosynthesis,	1.09
**57.**	**b0525**	ppiB	accelerates protein folding; chaperone activity and plays a role in bacterial cell division.	1.04

Supplementary Table 4: List of essential genes downregulated upon overexpression of $YxiD^{CTD}$ toxin in *E. coli*.

**S. No.**	**Gene**	**Gene symbol**	**Function**	**log_2FoldChange**
**1.**	**b3974**	coaA	precursor for coaA biosynthesis	-1.39
**2.**	**b3850**	hemG	helps organize the nucleoid	-1.17
**3.**	**b3967**	murI	essential for biosynthesis of D-Glutamate; provides rigidity & protection to bacterial cell wall	-1.21
